# Sphingosine-1-Phosphate Lyase Deficient Cells as a Tool to Study Protein Lipid Interactions

**DOI:** 10.1371/journal.pone.0153009

**Published:** 2016-04-21

**Authors:** Mathias J. Gerl, Verena Bittl, Susanne Kirchner, Timo Sachsenheimer, Hanna L. Brunner, Christian Lüchtenborg, Cagakan Özbalci, Hannah Wiedemann, Sabine Wegehingel, Walter Nickel, Per Haberkant, Carsten Schultz, Marcus Krüger, Britta Brügger

**Affiliations:** 1 Heidelberg University Biochemistry Center, Heidelberg, Germany; 2 European Molecular Biology Laboratory, Heidelberg, Germany; 3 CECAD, Cologne, Germany; Medical University of South Carolina, UNITED STATES

## Abstract

Cell membranes contain hundreds to thousands of individual lipid species that are of structural importance but also specifically interact with proteins. Due to their highly controlled synthesis and role in signaling events sphingolipids are an intensely studied class of lipids. In order to investigate their metabolism and to study proteins interacting with sphingolipids, metabolic labeling based on photoactivatable sphingoid bases is the most straightforward approach. In order to monitor protein-lipid-crosslink products, sphingosine derivatives containing a reporter moiety, such as a radiolabel or a clickable group, are used. In normal cells, degradation of sphingoid bases via action of the checkpoint enzyme sphingosine-1-phosphate lyase occurs at position C2-C3 of the sphingoid base and channels the resulting hexadecenal into the glycerolipid biosynthesis pathway. In case the functionalized sphingosine looses the reporter moiety during its degradation, specificity towards sphingolipid labeling is maintained. In case degradation of a sphingosine derivative does not remove either the photoactivatable or reporter group from the resulting hexadecenal, specificity towards sphingolipid labeling can be achieved by blocking sphingosine-1-phosphate lyase activity and thus preventing sphingosine derivatives to be channeled into the sphingolipid-to-glycerolipid metabolic pathway. Here we report an approach using clustered, regularly interspaced, short palindromic repeats (CRISPR)-associated nuclease Cas9 to create a sphingosine-1-phosphate lyase (*SGPL1*) HeLa knockout cell line to disrupt the sphingolipid-to-glycerolipid metabolic pathway. We found that the lipid and protein compositions as well as sphingolipid metabolism of *SGPL1* knock-out HeLa cells only show little adaptations, which validates these cells as model systems to study transient protein-sphingolipid interactions.

## Introduction

Cell membranes contain hundreds to thousands of individual lipid species and a multitude of proteins [1, 2]. Sphingolipids (SP), like the other major membrane lipid categories glycerolipids (GL), glycerophospholipids (GP) and sterols (ST) [3, 4], constitute a complex and highly versatile class of lipids involved in building membrane structures and membrane domains [5]. In addition, SPs and their metabolites [e.g. sphingosine (Sph), sphingosine-1-phosphate (S1P) and ceramide (Cer)] play important roles in intra- and extracellular signaling pathways such as the regulation of cell proliferation, apoptosis and intracellular trafficking [6].

Synthesis of complex sphingolipids is initiated by N-acylation of the sphingoid base, which is catalysed by a set of six ceramide synthases with remarkable fatty acid specificity and specific tissue distribution [7], which highlights the importance of the individual lipid species and their fatty acid length in particular [8]. Also within the membrane, SPs show specific interaction with and functional regulation on proteins [9, 10].

To study these and similar interactions bifunctional lipids are powerful tools [11, 12], which e.g. can combine a photoactivatable group with an alkyne or azide group that can be used in click chemistry [13]. To function in lipids both groups have to be small and hydrophobic to ensure they behave like their endogenous counterparts. Therefore, commonly used functionalities for lipids are the diazirine group and the alkyne group [14]. Photoactivatable and clickable sphingosine (pacSph, S1 Fig) is a promising new addition to the tool set, as it enables to specifically crosslink sphingolipids to interacting proteins via the diazirine group and provides an additional click functionality to allow for detection by adding a fluorophore or enrichment by adding a tag for affinity purification [11, 15].

However, while radioactively labeled photo-sphingosine [16] was designed in a way that the label is lost upon sphingosine degradation, pacSph’s photoactivatable and clickable group contained in the sphingoid base hydrocarbon chain are maintained after degradation to hexadecenal and subsequent oxidation and activation to palmitoyl-CoA [17–19]. As fatty acyl-CoAs are basic building blocks for glycero- and glycerophospholipids, specific sphingolipid labeling is quickly lost (S2 Fig). This is also true for any other sphingoid base containing a modification (e.g. a fluorescent dye) in its backbone [19, 20].

Draining of sphingolipid metabolic labeling into other pathways can be prevented by targeting the sphingolipid-to-glycerolipid metabolic pathway [17, 18]. Sphingosine-1-phosphate lyase (SGPL1) [21–23] is the first enzyme of this pathway and responsible for the irreversible breakdown of S1P at the C2-C3 carbon-carbon bond, resulting in formation of ethanolamine phosphate and the long chain aldehyde hexadecenal [17].

Mouse embryonic fibroblasts (MEFs) derived from homozygous sphingosine-1-phosphate lyase 1 (*Sgpl1*)-null mouse embryos (*Sgpl1*^−/−^) [24] exist and can be used for metabolic labeling with pacSph [15].

In order to allow for analysis of protein-sphingolipid interactions in other species, such as human, we performed a gene knock out of the *SGPL1* gene in HeLa cells by using the clustered regularly interspaced short palindromic repeat (CRISPR) type II system of *S*. *pyogenes* [25–27]. This was accomplished by using the CRISPR-associated RNA-guided endonuclease Cas9 [28, 29], which allows site-specific editing of genomes. CRISPR/Cas9 introduces double strand breaks at the target site, which can be repaired via the endogenous error-prone nonhomologous end-joining (NHEJ) repair pathway, leading to small deletions or insertions [30]. NHEJ repair at the initial exons of the gene can lead to frame shifts and premature stop codons. We chose to use a knock-out as compared to a knock down (e.g. RNAi) strategy as residual enzyme activity might still have enough capacity to keep metabolites within normal levels [31]. Here we characterize a *SGPL1* knockout HeLa cell line as a tool for studying protein-lipid interaction using bifunctional sphingolipid precursors.

## Materials and Methods

### Materials

pacSph was kindly provided by Per Haberkant and Carsten Schultz (EMBL, Heidelberg). pacFA GlcCer [D-glucosyl-ß-1,1' N-(9-(3-pent-4-ynyl-3-H-diazirin-3-yl)-nonanoyl)-D-*erythro*-sphingosine, #900405, Lot: PACFAGLCCER-10], pacFA Ceramide [N-(9-(3-pent-4-ynyl-3-H-diazirin-3-yl)-nonanoyl)-D-*erythro*-sphingosine, #900404, Lot: PACFACER-10], pacFA-18:1 PC [1-(9-(3-pent-4-ynyl-3-H-diazirine-3-yl)-nonanoyl)-2-oleoyl-*sn*-glycero-3-phosphocholine, #900408, Lot: PACFA-181PC-10] and D-*erythro*-sphingosine (#860020P) were purchased from Avanti Polar Lipids (Alabama, USA). All solvents were HPLC grade and purchased from Sigma-Aldrich. 3-azido-7-hydroxycoumarin (coumarin azide CLK-FA047-1) was purchased from Jena Bioscience. IncuCyte^™^ Kinetic Caspase-3/7 Apoptosis Assay Reagent (Cat No 4440) was purchased from Essen Bioscience.

### Antibodies

Anti-DDDDK tag (anti-Flag) antibody (abcam: ab1162, LOT: GR154017-4), anti-rabbit antibody IRDye^®^ 800CW (Rockland Immunochemicals Inc., 611-131-002), rabbit anti-calnexin (stressgen, SPA-865), goat anti-mouse IgG Secondary Antibody, Alexa Fluor^®^ 680 conjugate (Life Techn., A-21057), rabbit sphingosine 1-phosphate lyase 1 Antibody (Thermo Scientific, PA1-12722), rabbit anti-sphingosine 1-phosphate lyase H-300 (Santa Cruz Biotechnology, sc-67368). Mouse anti-CerS6, LASS6 monoclonal antibody (Abnova, H00253782-M01).

### Plasmids

pSpCas9(BB)-2A-GFP (PX458) was a gift from Feng Zhang (Addgene plasmid # 48138). *Homo sapiens* STARD7, Myc-DDK-tagged (STARD7-FLAG) (OriGene Technologies, Inc., Rockville, MD, #RC202539-OR). FLAG-p24 was generated (GeneArt, Regensburg, Germany) and inserted into pCMV6 (OriGene Technologies, Inc., Rockville) with KpnI and NotI restriction sites.

### Cell lines

Murine embryonic fibroblasts (MEFs) of wild-type (*Sgpl1*^+/+^) and *Sgpl1*-deficient (*Sgpl1*^−/−^) embryos were kindly provided by Paul P. Van Veldhoven [24]. MEFs were cultured in Dulbecco’s Modified Eagle Medium (DMEM, 4.5 g/l glucose) with 10% fetal calf serum (FCS, Sigma 7524), penicillin/streptomycin (Biochrom, A 2213), glutamine (Biochrom, K0283), non-essential amino acids (Biochrom, K 0293) and sodium pyruvate (Biochrom, L 0473) at 37°C with 5% CO_2_.

HeLa (ATCC) and HeLa ΔS1PL cells were cultured in Minimum Essential Medium Eagle with Alpha Modification (αMEM, Sigma M8042) supplemented with 10% FCS (Sigma 7524), penicillin/streptomycin and glutamine at 37°C with 5% CO_2_.

### CRISPR mediated knock out of the *SGPL1* gene in HeLa cells

HeLa Δ*SGPL1* were engineered according to [29]. In short: sgRNA guide sequences were either published earlier [27] or designed with online tools [32]. Oligos (Table 1) were synthesized (Biomers, Ulm, Germany) such that the annealed double strand could be cloned into the BbsI site of the pSpCas9(BB)-2A-GFP vector (Plasmid received from Feng Zhang, Broad Institute, Cambridge via Addgene: PX458).

**Table 1:** Oligonucleotides inserted into the BbsI site of pSpCas9(BB)-2A-GFP. sgRNA sequences are indicated in bold and preceded by a G nucleotide to improve transcription [29].

sgRNA	Orientation	Sequence
S1	fwd	CACCG**AGATGAGCTCAGACCCTGGG**
S1	rev	AAAC**CCCAGGGTCTGAGCTCATCT**C
S2	fwd	CACCG**AATCTCTAAGTAGGGCTCAA**
S2	rev	AAAC**TTGAGCCCTACTTAGAGATT**C
S3	fwd	CACCG**TAATTGCATGGAGTGTCGTG**
S3	rev	AAAC**CACGACACTCCATGCAATTA**C
S4	fwd	CACCG**GCCTCTATCTTGCGTAGTCC**
S4	rev	AAAC**GGACTACGCAAGATAGAGGC**C

pSpCas9(sgRNA) plasmids were confirmed by sequencing and introduced into HeLa cells using FuGENE HD as a transfection reagent according to the instructions of the manufacturer (Roche). After 3 days single cells positive for GFP were isolated by fluorescence-activated cell sorting and individual clones were expanded to cell lines. Clonal cell lines were verified via metabolic labeling with pacSph, reaction with coumarin azide and TLC analysis. For later experiments several clones were combined to compensate for clonal effects: sgRNA:S1 clone 1; sgRNA:S2 clone 3; sgRNA:S3 clones 0, 1 & 4 and sgRNA:S4 clone 0.

### Sequencing

Genomic PCR was run with the following primers: Forward primer: AAGAAGGGCAAGATCCGGAA, reverse primer: GTGGATCACGAGGTCAAGAGA using the TITANIUM Taq PCR Kit (Takara, 639210). Sequences were subcloned into pCR^™^2.1 Vector (Life technologies, TA Cloning^®^ Kit, K2020) and individual clones were sent for sequencing using the M13-FP primer: TGTAAAACGACGGCCAGT (GATC Biotech).

### Metabolic labeling

Cells were metabolically labeled with bifunctional sphingosine (S1 Fig). For labeling, cells were grown with 6 μM pacSph for the times indicated in media with delipidated FCS (Gibco 12676–011). pacSph was stored as a 25 mM ethanolic stock solution at -20°C.

### Lipid extraction for thin layer chromatography

Cells were scraped into 1 ml cold PBS, transferred to a 1.5 ml Eppendorf tube, pelleted (1,000 g, 5 min at 4°C) and resuspended in 300 μl cold PBS. To the suspension 600 μl methanol and 150 μl CHCl_3_ were added, each followed by vortexing.

The precipitated protein was pelleted (14,000 g, 5 min, RT). If the protein was used for SDS-PAGE, it was solubilized with 5 μl 10% SDS and then diluted in 200 μl lysis buffer (1×PBS, 1% Triton X-100, 0.1% SDS). Protein concentrations were determined with the Pierce^™^ BCA Protein Assay Kit (Pierce, 23225).

The organic supernatant containing the lipids was transferred to a 2 ml tube. Two phases were induced by the addition of 300 μl CHCl_3_ and 600 μl 0.1% acetic acid in H_2_O. After vigorous vortexing, the phases were separated by centrifugation (14,000 g, 5 min, RT) and the lower phase was transferred to a new 1.5 ml Eppendorf tube, in which the solvent was evaporated in a speedvac at 30°C.

### Saponification

Lipid extracts were dissolved in 1 ml 0.2 M NaOH in MeOH (control samples in 1 ml MeOH) and incubated for 120 minutes at 30°C. The saponified sample was neutralized with 8 μl glacial acetic acid and the lipids were re-extracted by adding 1 ml CHCl_3_ and 2 ml H_2_O. Phases were separated for 2 min at 20000 g and the organic phase was collected and dried in a speedvac.

### Thin layer chromatography of labeled cells

After metabolic labeling cells were analyzed as previously described [14, 33]. For coumarin TLCs cells were grown in 6-cm dishes and extracted for TLC as described above. Lipids were resuspended in 10 μl CHCl_3_ and 30 μl of a freshly prepared click mix: 2.5 μl of 44.5 mM 3-azido-7-hydroxycoumarin (coumarin azide, Jena Bioscience, CLK-FA047-1), 250 μl of a 10 mM Tetrakis(acetonitrile)copper(I) tetrafluoroborate (Sigma, #677892) in acetonitrile and 1 ml ethanol). The mixture was vortexed and incubated at 42°C in a thermoblock until the solvent was completely condensed under the lid. The sample was spun and applied to a 20 x 20 cm TLC Silica gel 60 (Merck, #1.05721.0001) using a 10 μl Microdispenser (Drummond: 3-000-210). TLC plates were developed using first CHCl_3_/MeOH/H_2_O/AcOH 65:25:4:1 for 50% of the plate and then hexane/ethylacetate 1:1 for the full plate. Coumarin fluorescence was enhanced by spraying the plate with 4% (v/v) N,N-diisopropylethylamine (Sigma-Aldrich, 496219) in hexane. Fluorescently labeled lipids were visualized using a geldoc system (Peqlab, Infinity 300, UV illumination) and fluorescent bands were quantified using the FIJI software package [34].

Where indicated TLCs were run according to a protocol developed by the Schultz laboratory. Cells were grown in a 12well format. Cells were washed twice with PBS, trypsinized for 5 min, pelleted (1,000 g, 5 min at 4°C) and taken up in 300 μl PBS. Lipids are extracted for TLC as described above and dissolved in 10 μl CHCl_3_ and 10 μl EtOH. To this 1 μl of TBTA (Sigma-Aldrich, 678937) solution (2.5 mM in DMSO), 10 μl of Tetrakis(acetonitrile)copper(I) tetrafluoroborate (10 mM in acetonitrile) and 1 μl of fluorogenic fluorescein solution (para-azido-fluorescein, 1 mM in EtOH [35]) was added. The reaction was transferred to a speedvac and evaporated at 45°C for 20 min. Lipids were dissolved in 30 μl CHCl_3_/MEOH/H_2_O/AcOH 65:35:4:1 of which 8 μl was spotted on a HPTLC Silica gel 60 (20x10 cm, Merck 1.05641.0001). Spotted TLCs were dried in a desiccator, developed to 7 cm with CHCl_3_/MEOH/H_2_O/AcOH 65:35:4:1, dried again (5 min), and developed to 9 cm with CHCl_3_/MEOH 8:1. The TLC was imaged with a blue (460 nm) epi light source of a Amersham Imager 600.

### Lipid synthesis

N-(Octadec-17-yn)-Sphing-4-enin-1-phosphocholine (click-SM) was synthesized as described before [36]: 5 mg (17.8 μmol) of 17-octadecynoic acid (Sigma-Aldrich, O8382) were dissolved under argon atmosphere in 4 ml of dry MeOH. 10.8 μmol (50 mg) D-*erythro*-sphingosylphosphorylcholine (M = 465.3 g/mol Avanti Polar Lipids, 860600P) in dry ethanol was added and the mixture was set to 45°C. 17 mg (68.8 μmol) of 2-ethoxy-1-ethoxycarbonyl-1,2-dihydroquinoline (EEDQ, Sigma-Aldrich, 149837) was added and the solution was stirred for 3.5 h. After another addition of 15 mg (60.7 μmol) of EEDQ and stirring for 45 min, the solution mixture was brought to RT and stirred for 16 h. The solvent was removed and the residue was purified by chromatography using silica gel 60 (0.063–0.200 mm, Merck, 1.07734.1000) and dichloromethane (25 ml), dichloromethane/MeOH (8:2, V/V, 25 ml) and dichloromethane/MeOH/H_2_O (50:25:6, v/v, 130 ml). Yield was 69%, (7.45 μmol, 5,4 mg, M: 727.57 g/mol). Mass was confirmed by precursor ion scan for m/z 184 on a triple-quadrupole-instrument (Quattro II, Micromass) as [M+H]^+^ 727.34.

### Lipidomics Analysis

2x10^5^ HeLa or MEF cells were seeded per 6well plate and grown for 2 days as described above. Cells were washed 2x with 150 mM NH_4_HCO_3_ in water, collected in 1 ml 150 mM NH_4_HCO_3_, pelleted at 8000 g for 3 min, resuspended in 50 μl of 150 mM NH_4_HCO_3_, snap frozen and stored at -20°C.

Lipid analyses were performed on a QTRAP5500 (ABSciex) coupled to a Triversa NanoMate device (Advion) as described in Özbalci et al 2013 [37]. Typically, ~1 nmol of total lipid was subjected to lipid extraction using an acidic Bligh&Dyer protocol [38], except for the analysis of plasmalogens (neutral extraction) and GM3 (2 step neutral extraction [39]. Standard lipid mixtures contained of 100 pmol PC (13:0/13:0, 14:0/14:0, 20:0/20:0; 21:0/21:0, Avanti Polar Lipids), 2, SM (d18:1 with N-acylated 15:0, 17:0, 25:0, semi-synthesized as described in [37]) and d6Chol (Cambrigde Isotope Laboratory), 55 pmol PI (16:0/16:0, 17:0/20:4, Avanti Polar Lipids), 50 pmol PE and PS (14:1/14:1, 20:1/20:1, 22:1/22:1, semi-synthesized as described in [37]), DAG (17:0/17:0, Larodan) and cholesterol ester (CE, 9:0, 19:0, 24:1, Sigma) 40 pmol TAG (D5-TAG-Mix, LM-6000 / D5-TAG 17:0,17:1,17:1, Avanti Polar Lipids), 10 pmol Cer and GlcCer (d18:1 with N-acylated 15:0, 17:0, 25:0, semi-synthesized as described in [37]), PA (PA 17:0/20:4, Avanti Polar Lipids) and PG (14:1/14:1, 20:1/20:1, 22:1/22:1, semi-synthesized as described in [37]). Plasmalogen (PE P-)-containing standard mix was supplemented with 33 pmol PE P-Mix 1 (16:0p/15:0, 16:0p/19:0, 16:0p/25:0), 46.5 pmol PE P- Mix 2 (18:0p/15:0, 18:0p/19:0, 18:0p/25:0), 64.5 pmol PE p- Mix 3 (18:1p/15:0, 18:1p/19:0, 18:1p/25:0), instead of PE. Semi-synthesis of PE P- was performed as described in [40]. Typically, 40 pmol GM3 (N-acylated CD3-18:0, Matreya) standard was spiked into 2 step neutral extractions. Data processing was performed using LipidView (ABSciex) and Microsoft Excel. GM3 and SM values of the HeLa Lipidome were measured from the 2 step neutral extractions from independent samples and adjusted to the rest of the lipidome by the average amount of the SM 34:1;2 species.

Quantitative analysis of sphingosine, sphinganine, sphingosine-1-phosphate and sphinganine-1-phosphate was performed following derivatization and LC-MS separation.

HeLa cells of a confluent 10cm dish, treated as described above, were resuspended in 200 μl 150 mM NH_4_HCO_3_ in water and transferred to a 2 ml-Eppendorf test tube containing 25 pmol of a sphingolipid standard mixture (LM-6002, with C17:1 bases of the various sphingoids; Avanti Polar Lipids) in 990 μl chloroform/methanol (17:1, v/v). Following a 120 min incubation 4°C on a thermomixer, samples were centrifuged for 5 min at 9000 g*av* and 4°C. The lower organic phase was transferred to a new tube, evaporated with nitrogen gas and dissolved in 110 μl methanol. To the remaining aqueous phase 10 μl of 2 M HCl was added, followed by 375 μl chloroform/methanol/37% HCl (40:80:1, v/v/v) and 125 μl chloroform. Samples were incubated for 120 min at 4°C on a thermomixer and then centrifuged as described above. The lower organic phase was transferred to a new 1.5 ml Eppendorf test tube and evaporated with nitrogen gas. Dried lipid extracts were dissolved in 110 μl methanol. A 10 μl of each phase was used for phosphate determination [37]. The remaining 100 μl of both the neutral and acidic extraction were subjected to TMS-diazomethane derivatization as described in [41]. Briefly, 10 μl of 2 M TMS-diazomethane in hexane was added to the solvent and the mix was incubated for 20 min at RT with 750 rpm/min shaking. The reaction was stopped by adding 1 μl acetic acid and evaporated under a gentle stream of nitrogen gas. Samples were resuspended in 100 μl isopropanol:water:acetonitrile (2:1:1) with 10 mM ammonium formiate and 0.1% formic acid. A 5 μl aliquot was subjected to mass spectrometric analysis. Samples were analyzed using a Dionex UHPLC coupled to a ThermoScientifc QExactive high-resolution mass spectrometer. UHPLC separations were performed using a CSH C18 column (100 x 2.1 mm ID, particle size 1.7 μm; Waters Corporation, Milford, MA, USA) as described [42] except for the LC conditions. Briefly, the gradient was run with a flow rate of 0.4 ml/min, starting with 10% solvent B (isopropanol/acetonitrile (90:10, v/v) with 10 mM ammonium formate and 0.1% formic acid), proceeding to 50% solvent B within 4 minutes, and then to 100% solvent B within 0.5 min. After 3.5 min at 100% solvent B, the gradient was reset to starting conditions with solvent A (acetonitrile/water (60:40) with 10 mM ammonium formate and 0.1% formic acid). Re-equilibration to 10% solvent B was done within 5 min. Detection and quantification of the targeted molecules carried out using the Xcalibur^™^ software.

For detection of pacSph labeled sphingomyelins HeLa Δ*SGPL1* cells were labeled with 3 μM pacSph for 6 h, extracted, saponified, re-extracted and measured as described earlier [43].

### Proteomics: Sample preparation and LC-MS/MS analysis

For the experiments control and Δ*SGPL1* HeLa cells were grown in standard MEM and 10% FCS until 90% confluency (~10E6 cells). Then, media were removed, cells were scraped and the cell pellets were immediately snap-frozen in liquid nitrogen. Protein extraction was performed with a lysis buffer containing (4% SDS, 100 mM Tris/HCL pH 7.6, 0.1 M DTT) and incubation at 95°C for 5 min. After sonication lysates were clarified by centrifugation at 16,000 g for 15 min at room temperature. To remove detergent lysates were precipitated with ice-cold acetone for 2 h at -20°C. After centrifugation at 4°C for 20 min, the pellet was washed twice with 80% ice-cold acetone. The protein pellet was dissolved in 6 M urea/2 M thiourea, 10 mM Hepes, pH 8.0 and the protein concentrations were determined by DC protein assay (Biorad). After alkylation with 55 mM iodoacetamide samples were incubated with 1 μg LysC for 2 h and after dilution with 4 volumes of 50 mM ammonium bicarbonate samples were further digested with 1 μg trypsin over night at room temperature. After stopping the reaction peptides were purified by stop and go extraction (STAGE) tips. Peptides were separated using a binary buffer system of A (0.1% (v/v) formic acid in H_2_0) and B (0.1% (v/v) formic acid in 80% acetonitrile) on an Easy nanoflow HPLC system (Thermo Fisher Scientific). We applied a linear gradient from 7 to 35% B in 220 min followed by 95% B for 10 min and then re-equilibration to 5% B for 10 min on a 50 cm column (75 μm ID) packed in-house with 1.9 μm diameter C18 resin. To control column temperature, we used a custom-made column oven at 40°C. The UHPLC was coupled via a nano-electrospray ionization source (Thermo Fisher Scientific, Bremen, Germany) to the quadrupole-based mass spectrometer QExactive Plus (Thermo Scientific, Bremen, Germany). MS spectra were acquired using 3e6 as AGC target at a resolution of 70,000 (200 m/z) in a mass range of 350–1650 m/z. A maximum injection time of 60 ms was used for ion accumulation. MS/MS events were measured in a data-dependent mode for the 10 most abundant peaks (Top10 method) in the high mass accuracy Orbitrap after HCD (Higher energy C-Trap Dissociation) fragmentation at collision energy 25 eV in a 100–1650 m/z mass range. The resolution was set to 17,500 at 200 m/z combined with an injection time of 60 ms. Statistical analysis was carried out with Perseus (Version 1.3.8.3). Significance Values are based on permutation based FDR analysis [44].

### Software

Digital images were processed using Photoshop and Illustrator (Adobe). Plots were created in R [45]. R packages used include ggplot2 [46], the bioconductor R package Gvis and others [47–49].

### Proliferation and apoptosis test

Cell proliferation and apoptosis experiments were performed employing an Essen BioScience IncuCyte Zoom live cell imaging microscope as described [50]. HeLa and HeLa Δ*SGPL1* cells were seeded in triplicates in 96well plates, applying 500, 1000, 2000 or 5000 cells per well. For apoptosis experiments, caspase reagent (Essen Bioscience) was added to the medium, resulting in a 1:1000 dilution of the reagent. As a positive control, apoptosis was induced by addition of 1 mM H_2_O_2_ to each well for the last 24 h of acquisition. Cell proliferation and apoptosis were monitored for 92 h, with 4 acquisitions per well every 4 hours. Data evaluation was performed using Excel.

### Protein click and western blot

HeLa or HeLa Δ*SGPL1* were seeded in 6wells (3x10^5^ cells in 3 ml medium/well) and grown for 24 h. At about 90% confluency, Flag-tagged candidate proteins were introduced as plasmids using FuGENE HD as a transfection reagent according to instructions of the manufacturer (Roche) except for the fact that only one third of the recommended DNA:Fugene complex was used (50 μl instead of 150 μl per 6well). Cells were then metabolically labeled with pacSph for 7 h. For competition experiments [15], cells were labeled with 0.5 μM pacSph with or without 5 μM sphingosine. Following labeling, cells were washed 2x with PBS and UV-irradiated (Sylvania R 100 W) in 500 μl of PBS for 5 min on an ice-cold metal block. After removal of PBS, cells were scraped into 1 ml of PBS, pelleted (16,000 g, 5 min) and lysed for 1 h in lysis buffer [50 mM HEPES-NaOH, pH 7.4, 100 mM NaCl, 1% Triton X-100 (v/v), 0.5% deoxycholate (w/v), and 2x protease inhibitor cocktail]. A postnuclear supernatant (PNS) was created by centrifugation (3,000 g, 8 min) and the supernatant was subjected to a click reaction protocol (modified from [51]): To 100 μl of PNS 3.25 μl of click mix was added adjusting the reaction to 500 μM CuSO_4_ (Sigma-Aldrich, 203165), 50 μM Alexa Fluor 647 Azide (Invitrogen, A10277), 500 μM Tris(2-carboxyethyl)phosphine (TCEP, Sigma, 646547) and 50 μM Tris[(1-benzyl-1H-1,2,3-triazol-4-yl)methyl]amine (TBTA, Sigma-Aldrich, 678937). The reaction was incubated for 5 h at RT in the dark.

From the reaction mixture Flag-tagged proteins were immunoprecipitated using EZview^™^ Red ANTI-FLAG^®^ M2 Affinity Gel (Sigma, F2426). After 1 h incubation at 4°C, immune complexes were washed with lysis buffer and proteins were eluted using SDS-PAGE sample buffer or a glycine plus FLAG peptide based elution. For the latter proteins were eluted with elution buffer [0.1 M glycine-HCl pH 3.5, 1% TritonX-100, 0.5% SDS, 0.5 g/l FLAG^®^ Peptide (Sigma, F3290)] immediately neutralized with 10% (v/v) of neutralization buffer [0.5 M Tris-HCl pH 7.5, 1.5 M NaCl] and precipitated with cold methanol at -80°C over night. The precipitated proteins were pelleted at 20000 g for 20 min at 4°C and taken up in SDS loading buffer [4x loading buffer: 100 mM Tris-HCl, pH 8.3, 50 mM DTT, 4 M Urea, 10% glycerol (v/v), 8% SDS (w/v), 0.01% bromophenol blue (w/v)], incubated for 5 min at 95°C and loaded on a 13% SDS-PAGE with Precision Plus Protein All Blue Standard (Bio-Rad 161–0373) as molecular weight marker. Proteins were transferred to a PVDF membrane (Millipore, IPFL00010). FLAG-antigens were detected with a rabbit anti-DDDDK tag antibody (abcam: ab1162, LOT: GR154017-4, 1:1000) and anti-Rabbit Antibody IRDye^®^ 800CW (Rockland Immunochemicals Inc., 611-131-002) using an infrared imaging system (Odyssey; LI-COR Biosciences) and software (Image Studio, Version 2.1.10).

Membrane proteins were prepared as follows: HeLa and HeLa Δ*SGPL1* cells were washed twice with PBS, scraped into 1.2 ml PBS and homogenized by 30 passages through a cold ball-bearing homogenizer (EMBL, Heidelberg, Germany) using the 8.010 mm ball. 0.2 μl of Benzonase (Sigma, E1014) were added and lysates were pulsed 3 times for 5 s in a sonicator bath. Samples were adjusted to 0.1 M Na_2_CO_3_ and 35% Iodixanol (Sigma, D1556) in a SW60 Tube. The mixture was overlaid with 1 ml of 30% Iodixanol in PBS and 0.5 ml of PBS, and membranes were floated in a SW60 rotor (Beckman Coulter) at 45 000 rpm/124740 g for 1 h. Equal protein amounts were precipitated with organic solvents [52] and immunoblotted as described above using 1:1000 mouse anti-LASS6 (Abnova, H00253782-M0) and 1:1000 rb anti-Calnexin (Stressgen, SPA-865).

## Results & Discussion

### Metabolism of pacSph in *Sgpl1*^+/+^ and *Sgpl1*^-/-^ cells

**Fig 1:**
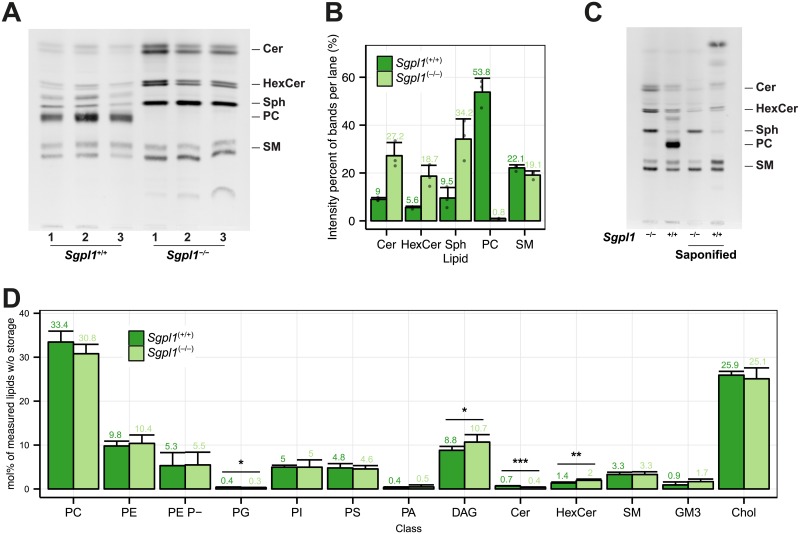
Characterization of the MEF *Sgpl1*^+/+^ and *Sgpl1*^-/-^ cell lines. (A) *Sgpl1*^+/+^ and *Sgpl1*^-/-^ cells were metabolically labeled with 6 μM pacSph for 4 h. Lipids were extracted and subjected to click reaction with fluorogenic coumarin azide, separated by TLC and exited with UV light. Lipids were identified via clickable standards (S3 Fig). Three replicates are shown for every cell line. (B) Quantification of the fluorescence intensities of Fig 1A. Values are listed in S1 Table. Mean ± SD are shown (n = 3). (C) Samples were treated as above, but were also subjected to alkaline hydrolysis (saponified) or mock treatment. (D) Membrane Lipidome. Lipid class profile of *Sgpl1*^+/+^ and *Sgpl1*^-/-^ cells. Lipid classes are standardized to all lipids measured excluding storage lipids (TAG and CE). GP O- lipids are contained in the sum of each class (e.g. PE O- in PE) and may show overlap with diacyl species containing odd-numbered fatty acids. PE Plasmalogens (PE P-) are displayed as a separate class. A Welch Two Sample t-test was used to estimate the P values: *P < 0.05; **P < 0.01; *** P < 0.001. Error bars correspond to standard deviation (n = 6).

To achieve exclusive incorporation of bifunctional pacSph into cellular sphingolipids, we aimed at disconnecting the sphingolipid-to-glycerophospholipid-hub by targeting sphingosine phosphate lyase activity [15]. To ensure that knocking out SGPL1 activity does not cause significant alterations in the cell’s metabolic state and its membrane lipid compositions we made use of mouse embryonic fibroblasts (MEFs) derived from either homozygous sphingosine-1-phosphate lyase 1 (*Sgpl1*)-null (*Sgpl1*^−/−^) or wild-type (*Sgpl1*^+/+^) mouse embryos [24]. To this end, cells were metabolically labeled with pacSph and, following extraction of cellular lipids, subjected to click reaction with fluorogenic coumarin azide [33] to allow for visualization of the fluorescent lipids separated by thin-layer chromatography (TLC) (Fig 1A). After 4 h of labeling about half of the pacSph was metabolized to the glycerophospholipid (GP) phosphatidylcholine (PC) in *Sgpl1*^+/+^ cells (Fig 1A and 1B). The PC band was identified by lipid standards (S3 Fig) and its susceptibility to mild alkaline hydrolysis (Fig 1C). Other saponifiable lipids and sphingolipids were also labeled, but to a lesser degree. In the Sgpl1 deficient *Sgpl1*^−/−^ cell line, on the other hand, only sphingolipids were labeled and PC or other saponifyable lipids were not observed. It is interesting to note that in *Sgpl1*^−/−^ cells specifically the short-chain sphingolipids (C16-C18), i.e. the slower migrating, lower band within ceramide, hexosylceramide and sphingomyelin [53], are stronger labeled than in *Sgpl1*^+/+^ cells. A preference for labeling of short-chain sphingolipids might be due to a partial inhibition of ceramide synthase 2 by S1P [54], which was reported to accumulate under SGPL1 knockout conditions [24]. In line with the fact that in *Sgpl1*^−/−^ cells S1P can only be metabolized by S1P phosphatase back to Sph a more efficient pacSph degradation in *Sgpl1*^+/+^ cells was observed, resulting in 9.5 ± 4.4% of quantified bands after 4 h. In *Sgpl1*^−/−^ cells the remaining pacSph is at about a third of the visualized lipids (34.2 ± 8.4%) (S1 Table). At the same time SP labeling in *Sgpl1*^−/−^ cells is increased compared to *Sgpl1*^+/+^ cells (Fig 1A). In summary and as expected, labeling of non-sphingolipids is blocked in *Sgpl1*^−/−^ MEF cells.

These results are consistent with a disruption of the sphingosine-1-phosphate (S1P) metabolic pathway of which SGPL1 represents the first step [18]. The pathway ends with palmitoyl-CoA, a basic lipid precursor, readily incorporated into GPs like PC [17, 18]. When *Sgpl1*^-/-^ cells are labeled with pacSph, which cannot be degraded in the absence of SGPL1, the lipid precursor can only be consumed by anabolic pathways of sphingolipid synthesis [55]. To rule out that disruption of the sphingolipid-to-glycerophospholipid pathway results in significant changes of cellular lipid homeostasis, which would exclude these cells as suitable models to study protein-sphingolipid interactions, we performed a quantitative mass spectrometric lipid analysis of *Sgpl1*^-/-^ and *Sgpl*^*+/+*^ cells (S1 Text).

### Lipid profiles of MEF *Sgpl1*^+/+^ and *Sgpl1*^-/-^ cells

For the analysis we grouped the lipids into functional categories (i.e. ST:sterols, SP:sphingolipids, GPL:[glycerophospholipids (GP) and the glycerolipid diacylglycerol (DAG)] and storage lipids:[Triacylglycerol (TAG) and cholesteryl ester (CE)]), which are derived from the LIPID MAPS categories [3, 4]. Both lipidomes showed no major difference on the lipid functional category level (S4A Fig). On the class level (Fig 1D) we found changes in the GPLs PG and DAG, with a 25% reduction in PG and a 20% increase in DAG in *Sgpl1*^-/-^ cells. Within the sphingolipids we observed a 40% reduction of ceramides in *Sgpl1*^-/-^ cells. This is contrast to the data by Coilé et al. who reported a 40% increase in total ceramides, when analysed using an enzymatic assay [24]. Compared to wild type mice, ceramide levels are also increased in the liver of *Sgpl1*^−/−^ mice [56] but no significant change is found in neuronal cultures of *Sgpl1*^−/−^ cerebella [57].

We found SM levels unchanged in *SGPL1*^*-/-*^ MEFs, however, cerebrosides (HexCer) increased by a factor of 1.4 (Fig 1D). While SM has been reported to increase in liver [56], no change in SM and HexCer has been observed in neurons [57]. Disruption of *Sgpl1* has different consequences in different tissues and cell types [24, 56, 57], and their installment of the complex network of enzymes and structural species involved in the creation of ceramide [6] and higher sphingolipids.

Sphingolipid length and hydroxylation profiles did not change significantly (S4C and S4F Fig), while SP species with 3 double bonds were slightly increased (S4E Fig). Individual species levels show no major changes (S5 Fig). *Sgpl1*^−/−^ lipids were shifted towards longer glycerophospholipids (GPL, 38 and 40 carbon atoms) drawing from lengths of 34 and 36 carbon atoms (S4D Fig). Consistent with longer fatty acids being usually more unsaturated, *Sgpl1*^−/−^ GPL double bonds are shifting towards more double bonds (4, 5 and 7 double bonds, S4G Fig). In summary, in *Sgpl1*^−/−^ GPL lipids [58] are slightly longer and SP and GPL have slightly more double bonds, however the overall changes are mild and do not compromise with the use of *Sgpl1*^−/−^ MEF cells as a tool to study protein-sphingolipid interaction using pacSph.

Currently only MEFs are available as immortalized cell line displaying dysfunctional SGPL1 activity. The fact that this restricts analyses towards one specialized cell type and one organism together with the fact that sphingolipid composition [7] vary dramatically in different contexts (organisms, organs, tissue and cells) [1], we sought out to develop a protocol for efficient and fast generation and validation of *SGPL1*^-/-^ cell lines. To this end we chose HeLa cells of human origin with the advantage of their high susceptibility towards transfection and growth in suspension culture.

### CRISPR KO of S1PL in HeLa cells

**Fig 2:**
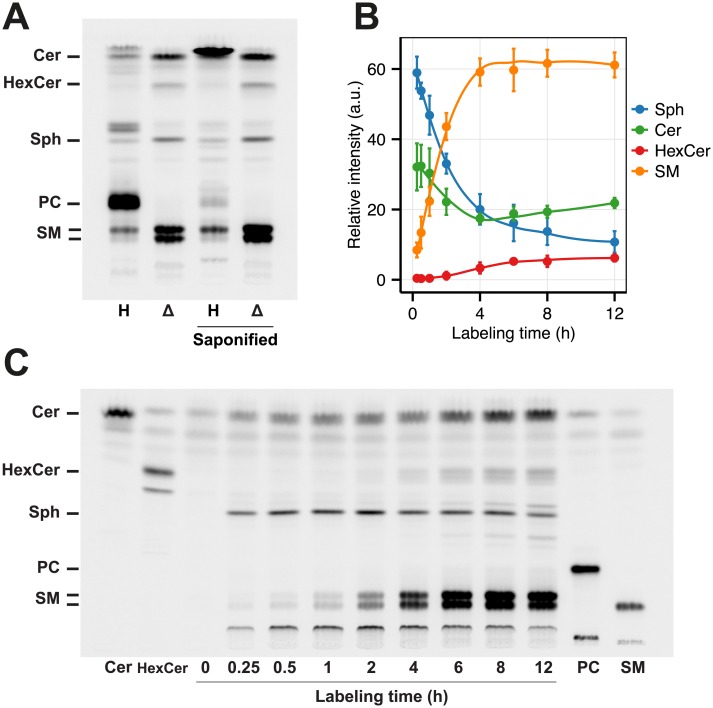
Characterization of the HeLa Δ*SGPL1* cell lines. (A) HeLa wildtype (**H**, Lane 1) and HeLa Δ*SGPL1* (**Δ**,Lane 2) cells were labeled with 6 μM pacSph for 6 h. Lipids were extracted and subjected to click reaction with fluorogenic fluorescein, separated by TLC and excited with blue light. Lipids were identified based on comparison to the respective lipid standards (see Fig 2C). Both samples were also subjected to alkaline hydrolysis (Lane 3 and 4). (B) Quantification of the fluorescence intensities of Fig 2C. Mean ± SD (n = 3) are shown (S2 Table). Each timepoint is standardized to 100% of lipids quantified. Data are fitted by Local Polynomial Regression Fitting (LOESS, degree = 2) as curves. (C) HeLa Δ*SGPL1* cells were labeled for the time points indicated and treated as in (A). As reference, 50 pmol of each clickable Cer, HexCer, PC and SM (S3 Fig) was loaded as standard.

To quickly create a cell line that can be easily handled (e.g. transfected) we established a CRISPR-associated RNA-guided endonuclease Cas9-mediated disruption of the *SPGL1* gene in HeLa cells. Plasmids expressing Cas9-GFP together with one of 4 sgRNA sequences [29] targeting a total of 3 exons of the *SGPL1* gene (S6 Fig) were used to transiently transfect HeLa cells. Individual clonal cell lines were isolated and screened for positive hits using pacSph metabolic labeling. Click reaction to fluorogenic coumarin azide and TLC analysis revealed that 70% (7 of 10) of the tested clones were lacking the prominent PC band apparent in wild type HeLa cells and were thus selected as positive (Fig 2A). When sequenced, reading frames were usually found disrupted in the genome by insertions or deletion (indel) mutations (S7 Fig). All sgRNA sequences used resulted in clones that were tested positive for a *SGPL1* knockout in the TLC assay. In the following a mixture of clones was used to compensate for clonal effects (see Materials and Methods).

We also successfully used this protocol to generate Δ*SGPL1* cells based on A549 human lung carcinoma cells and HEK cells.

In summary, the procedure proofed to be easy, fast and transferable to other human cell lines, allowing to generate a tool box based on cell lines reflecting different species and organ backgrounds to be used to study protein-sphingolipid interactions. Such cell lines in combination with HeLa cells deficient in genes involved in sphingolipid metabolism [31] then allows for analysis of sphingolipid protein interaction in specifically altered cellular lipid compositions.

Besides the first step of the sphingolipid-to-glycerophospholipid metabolic pathway (*SGPL1)*, we also attempted to knock out the fatty aldehyde dehydrogenase *ALDH3A2* (S8 Fig) [18, 59], which represents the second step in the pathway. However, we did not find any clones devoid of PC labeling after metabolic labeling with pacSph and TLC analysis. In this case we only targeted one exon, which could be one reason why the procedure did not work. Furthermore it is possible that in HeLa cells another subtype of the fatty aldehyde dehydrogenase could be involved in the sphingolipid-to-glycerophospholipid metabolic pathway or that the gene knock out would prevent the cells from surviving the procedure.

### Lipid profiles and Sphingosine Metabolism of HeLa Δ*SGPL1* cells

HeLa and HeLa Δ*SGPL1* cells were labeled with pacSph, subjected to click reaction with fluorogenic fluorescein azide [35] and analyzed by fluorescent TLC (Fig 2A). As in case of MEF cells HeLa wild type cells labeled with pacSph showed mainly incorporation of the alkyne moiety into PC. As expected, PC was susceptible to saponification, while sphingolipids were resistant. In contrast, no incorporation of the alkyne moiety into PC was observed in HeLa Δ*SGPL1* cells. Consistent with the metabolic labeling of *Sgpl1*^−/−^ MEF cells, sphingolipids in HeLa Δ*SGPL1* cells are labeled stronger and pacSph is degraded slower than in HeLa wildtype cells. Again we see a stronger labeling of short-chain SPs. Thus, as in *Sgpl1*^−/−^ MEF cells, HeLa Δ*SGPL1* pacSph labeling of HeLa Δ*SGPL1* results in exclusive labeling of sphingolipids.

#### Rescue of the *SGPL1* knockout

**Fig 3:**
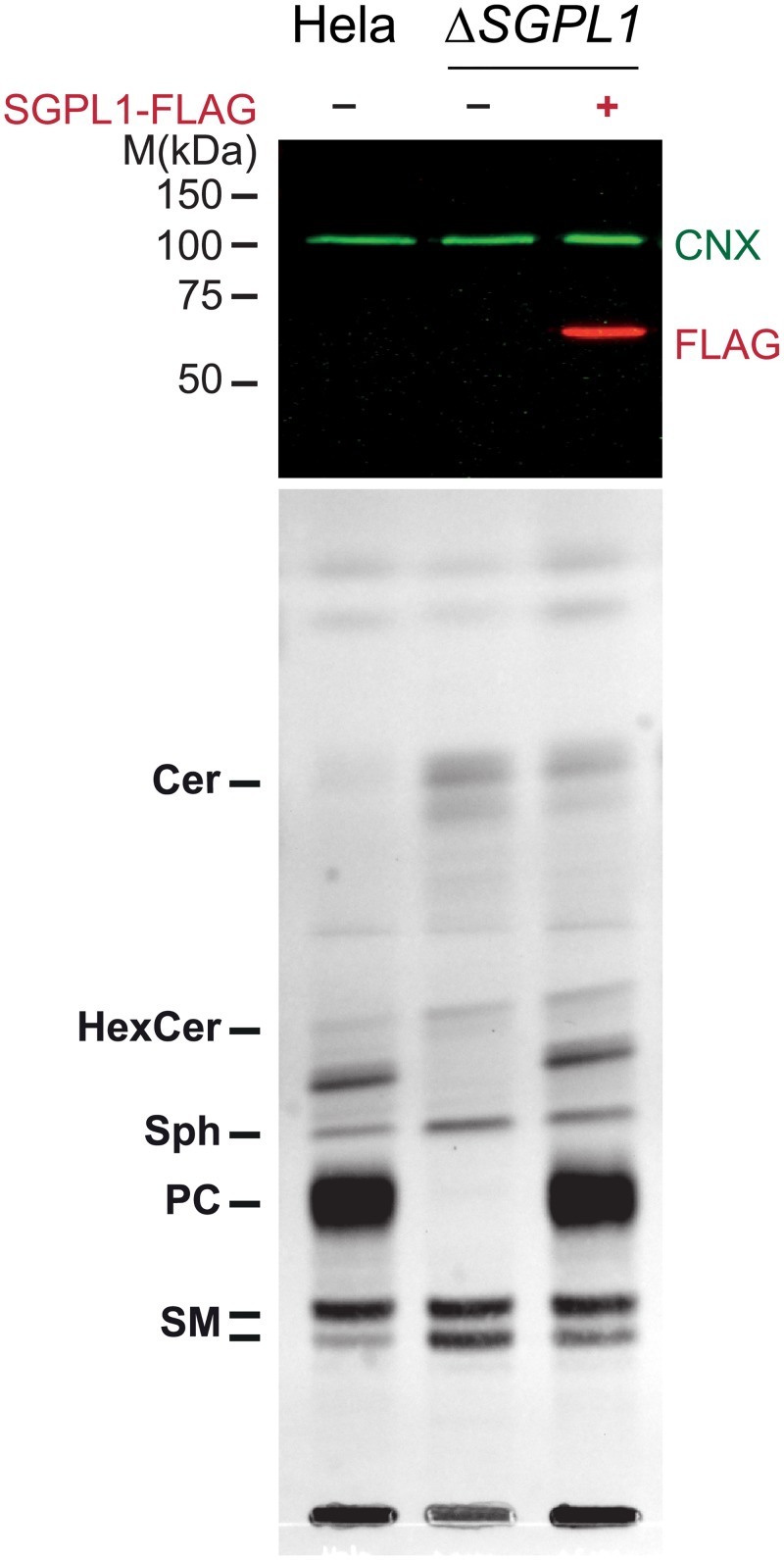
Rescue of HeLa Δ*SGPL1* cells by exogenous Flag-SGPL1. HeLa wildtype, HeLa Δ*SGPL1* and HeLa Δ*SGPL1* transfected with Flag-tagged SGPL1 were labeled with 6 μM pacSph for 4 h. **Upper Panel:** Proteins precipitated during the lipid extraction were solubilized, analyzed by SDS-PAGE and immunoblotting, using anti-FLAG antibodies (red). Detection of endogenous calnexin with anti-calnexin antibody (CNX, green) was used as a loading control. **Lower Panel:** Extracted lipids were subjected to click reaction with fluorogenic coumarin azide, separated by TLC and exited with UV light. Lipids were identified via with alkyne lipid standards (S3 Fig).

Although there are commercial SGPL1 antibodies, none of the antibodies we tested detected endogenous levels of the SGPL1 protein in HeLa cells. Therefore, we exogenously expressed a Flag-tagged version of SGPL1 in HeLa Δ*SGPL1* cells (Fig 3 upper panel). Although the *SGPL1* coding region on the plasmid would be a target for Cas9 loaded with the sgRNA we provided, transient expression of Cas9 had already faded at the time of the experiment. pacSph labeling of HeLa wild type cells resulted in the characteristic strong PC labeling, which was abolished in HeLa Δ*SGPL1* cells (Fig 3 lower panel). Exogenous expression of Flag-tagged SGPL1 completely reverted that phenotype and strong PC labeling was observed again. Thus, the *SGPL1* knockout strain could be completely rescued by exogenously expressed SGPL1 protein. These results further strengthen a successful knockout of the *SGPL1* gene as the cause of the altered labeling pattern in HeLa Δ*SGPL1* cells.

#### Metabolism of pacSph in HeLa ΔSPGL1 cells

Following the metabolization of pacSph in HeLa Δ*SGPL1* cells over a time course of 12 h shows roughly constant levels of pacSph for most of the time, while all sphingolipid classes derived from the pacSph precursor accumulate the label gradually (Fig 2C). Standardizing intensities the total intensity within each time point yields a more differentiated picture (Fig 2B). pacSph is metabolized continuously from 60% of quantified bands after 15 min to 10% after 12 h, while SM reaches a plateau at 60% after the first 4 h (S2 Table). Ceramide labeling is initially high (30%) and drops as it is further consumed. After 4 h its relative levels raise again to a final of 20% of label. Hexosylceramides, however, are steadily increasing over the total time course reaching 6% of label after 12 h. According to these data, time points can be chosen to preferentially label either ceramide or more complex sphingolipids (S3 Table).

#### HeLa and HeLa Δ*SGPL1* lipid profiles

**Fig 4:**
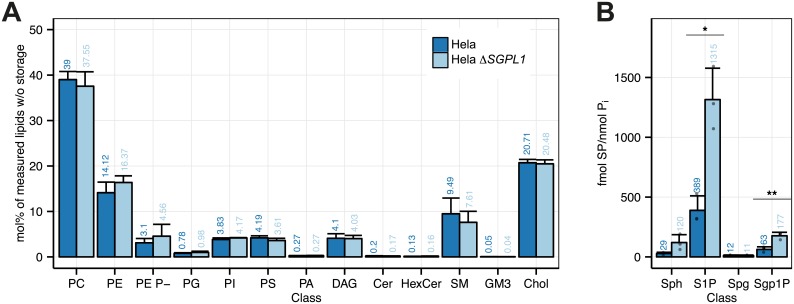
Lipidomics analysis of the HeLa and HeLa Δ*SGPL1* cell lines. (A) Class profile, standardized to all lipids measured without storage lipids (TAG and CE). GP O- lipids are contained in the sum of each class (e.g. PE O- in PE) and may show overlap with diacyl species containing odd-numbered fatty acids. PE P-lipids are displayed as a separate class. (B) Sphingoid bases and sphingoid base 1-phosphates standardized to total phosphate (fmol/nmol P_i_). Data are shown in S3 Table. A Welch Two Sample t-test was used to estimate the P values: *, p < 0.05; **, p < 0.01; ***, p < 0.001. Error bars correspond to standard deviation (n = 3).

HeLa and HeLa Δ*SGPL1* lipid profiles (S2 Text) are very similar concerning the functional categories (S9A Fig) and the lipid classes (Fig 4). In HeLa Δ*SGPL1* sphingolipids are slightly shorter (S9C Fig) and less saturated (S9E Fig). GPLs with 40 carbon atoms are more and with 36 carbon atoms less abundant (S9D Fig), while there are slightly more GPLs with 4 and 5 double bonds (S9G Fig) and changes within the sphingolipid species are also quite mild (S10 Fig). Sphingosine and S1P have been found strongly increased in both liver tissue and neural cells from *SGPL1* deficient mice [56, 57]. Here we find moderate three-fold elevate levels in S1P and sphinganine-1-phosphate (Sgh1P). Sphingosine, and sphinganine do not to change significantly (Fig 4B, S3 Table).

S1P acts as an intracellular second messenger [60] as well as a ligand for ubiquitously expressed G protein-coupled receptors on the cell surface [61, 62], thereby controlling cell migration, proliferation and programmed cell death pathways [63]. In most cell types, S1P and ceramide have antagonistic effects on cell survival, with S1P as proliferative stimulus and ceramide as activator of cellular stress responses and apoptosis [64]. However, S1P responses are cell-type specific [65, 66]. While strong effects on the lipidome were observed in mouse liver tissues and serum [56], little changes were seen in neurons [57]. The latter are explained by the reduced *de novo* long chain base biosynthesis and a corresponding increase in the recycling of backbones via the salvage pathway. Here we find that disrupting the *SGPL1* gene does not have a strong influence on the lipidome of HeLa cells, making them a useful model cell line in protein-lipid interaction studies.

### Comparison of the lipidome changes in MEF and HeLa cells upon *SPGL1* knock-out

Lipidomes of MEF and HeLa cells are different in terms of absolute values. We asked if the disruption of the *SGPL1* gene has similar effects on both lipidomes. To this end we calculated the relative change upon disruption of the *SGPL1* gene (See equations at S11 Fig). The relative change is characterized by a direction (positive for increase from e.g. HeLa to HeLa Δ*SGPL1*) and a magnitude of relative change. We found that the responses of the two cell types are quite heterogeneous (similar and dissimilar) for categories, classes and sphingolipid features (S11 and S12 Figs), which therefore do not reflect a coordinated reaction of both cells. However, with respect to changes in the level of length and double bonds in GPL lipids both cell types overall react in the same direction and similar magnitude (S12D and S12G Fig). Therefore MEF and HeLa lipidomes respond individually and specific to the disruption of the *SGPL1* gene, with the exception that changes of GPL in HeLa and MEF cells revealed similar trends with respect to their direction and magnitude.

### HeLa Δ*SGPL1* proteome analysis

**Fig 5:**
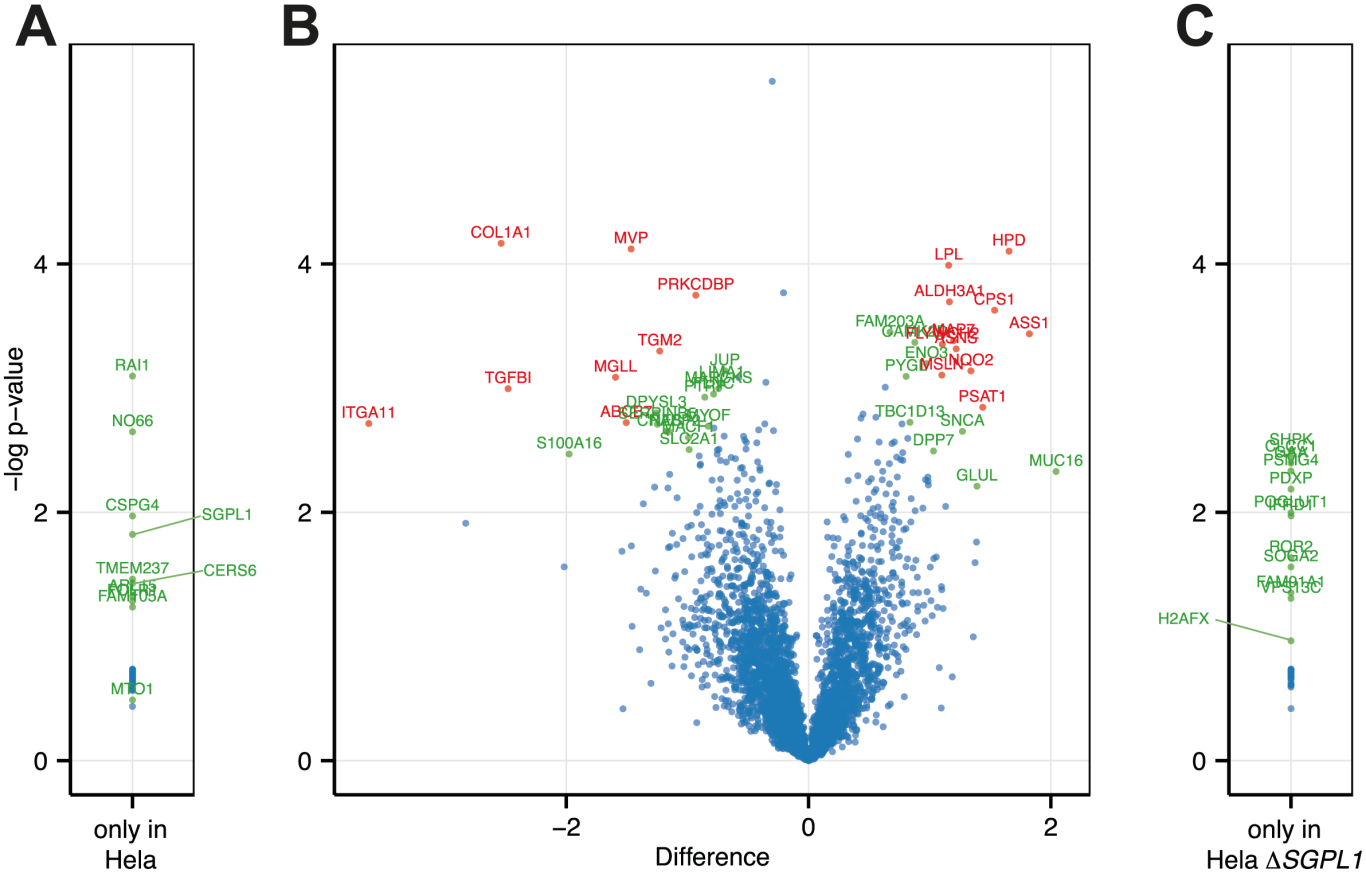
Proteomic analysis of the HeLa and HeLa *ΔSGPL1* cell lines. Differential expression of proteins in HeLa and HeLa Δ*SGPL1* cell lines. **(A and C)** Proteins only found in one of the cell lines. Protein LFQ (label free quantification) intensities of HeLa (A) or HeLa Δ*SGPL1* (C) cells were used to calculated T-test p-values. A list of proteins is provided in S5 and S6 Tables. **(B)** Volcano plot of differential expression of proteins. Difference of log transformed LFQ (label free quantification) intensities of HeLa cells and HeLa Δ*SGPL1* cells (Δ*SGPL1* –HeLa). Statistical analysis was carried out with Perseus software. Significance Values are based on permutation based FDR analysis [44]: Proteins scoring p < 0.05 are shown in green, p < 0.01 in red. A list of proteins is provided in the S4 Table.

As *SGPL1* gene disruption can cause widespread changes in cellular expression patterns of lipid metabolism genes [56] and possible further genes, we subjected HeLa and HeLaΔ*SGPL1* to proteome analysis. We found only 41 significant changes within the 3539 proteins identified (Fig 5B and S4 Table). In addition, 10 proteins were only found in HeLa cells (Fig 5A and S5 Table) and 12 only in HeLa Δ*SGPL1* (Fig 5C and S6 Table). As expected, SGPL1 was only found in HeLa and not in HeLa Δ*SGPL1*. On the other hand the histone variant H2AX a is found only in HeLa Δ*SGPL1*, which is consistent with its role in efficient repair of DNA double strand breaks [67], presumably caused in the process of Cas9 mediated genome editing [68]. When we compare our dataset to the homolog dataset obtained by microarray analysis of lipid metabolism genes in liver [56] we find no overlap of proteins (except *SGPL1*) that would be significantly changed in both studies. A surprising finding is the seeming complete loss of Ceramide synthase 6 (CerS6) expression in HeLa Δ*SGPL1* cells. This would be rather surprising as CerS6 creates ceramides with C16 fatty acids [7] and we see an overall increase in ceramides with 34 carbon atom, which should contain C16 fatty acids (S9C and S10 Figs). However, CerS5 which is also producing C16 fatty acid ceramides could potentially compensate [7]. An immunoblot for CerS6 did show CerS6 clearly present in HeLa Δ*SGPL1* cells (S13 Fig). Therefore, the CerS6 hit should be a false positive. Further analysis including quantitative determination of mRNA levels will help to evaluate the significance of the protein hits identified and to investigate if the changes found for 1% of total protein are in response to the loss of the *SGPL1* gene.

In addition to the proteome analyses we compared proliferation and apoptosis in Hela and HeLa Δ*SGPL1* cells and did not find a significantly different behaviour of these two cell lines (S14 Fig).

Taken together we did not see major changes in the proteome, the cell proliferation and the apoptotic activity induced by the disruption of the *SGPL1* gene, which would hamper the use of the cell line for lipid protein interaction studies.

### Analysis of protein-lipid interaction in HeLa Δ*SGPL1* cells

In the experiments described above, we used the alkyne group of pacSph together with click reaction mediated fluorescent labeling of the lipid. The photoactivatable diazirine group (S2 Fig) enables the specific cross-linking of metabolically labeled sphingolipids with its interacting proteins upon UV radiation [11]. The presence of a lipid cross-link to a protein can then again be visualized by using the alkyne functionality in click reaction with a fluorescent dye. When combined in western blot analysis different fluorescent dyes for detection of the protein and the lipid have to be used. HeLa Δ*SGPL1* cells can now be used as a tool for specifically studying protein-sphingolipid interaction as pacSph is only incorporated into sphingolipids. Identification of the sphingolipid bound to a protein of interest can be achieved by mass spectrometric characterization of lipids bound to the full length protein or to peptides following protease digest. Mass spectrometric analysis of protein-lipid crosslink products usually is a challenging task. A first indication on the nature of the bound sphingolipid can be obtained by interfering with the metabolism of pacSph, i.e. by using inhibitors specifically blocking one step of sphingolipid biosynthesis such as Fumonisin B1, which inhibits ceramide synthases or D,L-*threo*-1-phenyl-2-palmitoylamino-3-morpholino-1-propanol (PPMP) as inhibitor of glucosylceramide synthase.

**Fig 6:**
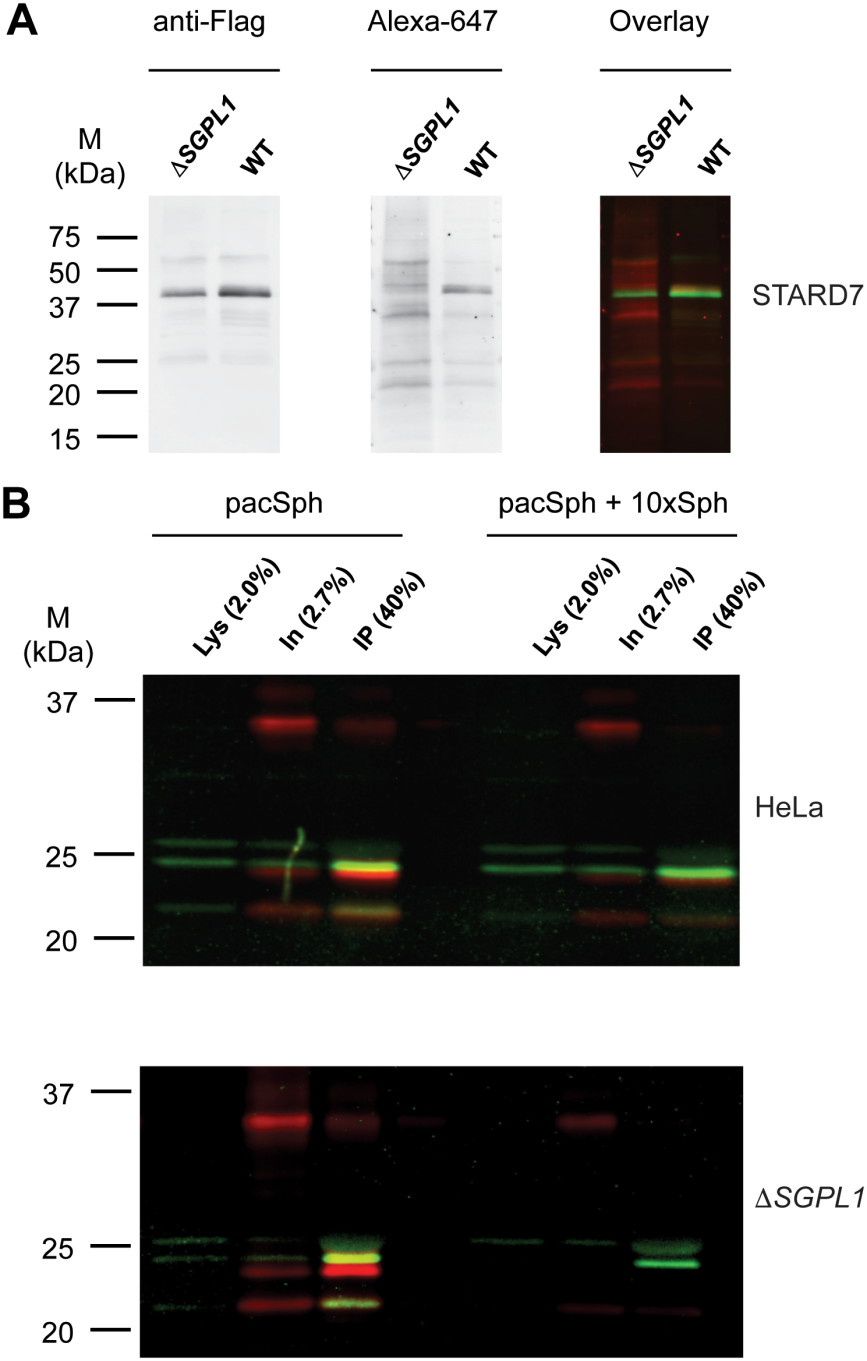
Fluorescent labeling of pacSph metabolites to study protein lipid interaction. Flag-tagged STARD7 (A) and p24 (B) were expressed in HeLa and HeLa Δ*SGPL1* cell lines. Cells were metabolic labeled with 5 μM pacSph (STARD7) or 0.5 μM pacSph with or without sphingosine (p24) for 7 h and then UV irradiated to cross-link pacSph metabolites to nearby protein. Protein lysates were subjected to click reaction with Alexa647 azide (shown in red) and the ectopically expressed proteins were immunoprecipitated. After SDS-PAGE and immunoblot with fluorescently labeled secondary antibodies (shown in green), lipid and protein signals were detected in separate channels. Lys, Lysate, IN, input of immunoprecipitation, IP, immunoprecipitated material.

We used STARD7, a START domain-containing protein that extracts PC, but not SM, from the cytoplasmic surfaces and delivers it to mitochondria [69, 70] to test for the specificity of sphingolipid labeling of candidate proteins. HeLa and HeLa Δ*SGPL1* were transfected with Flag-tagged STARD7, metabolically labeled with pacSph and UV-radiated. Cell lysates were subjected to click reaction with an Alexa647 fluorescent dye. Immunoprecipitated Flag-STARD7 was visualized by immuoblot using fluorescent secondary antibodies (Fig 6A). A protein-lipid crosslink product was only found in HeLa but not in HeLa Δ*SGPL1* cells. This is consistent with the presence of pacSph derived pacPC in HeLa cells, while HeLa Δ*SGPL1* cells are devoid of pacPC (Fig 2).

STARD7 is a cytosolic PC binding protein with high specificity [70], but most protein-lipid interactions occur within cellular membranes where membrane proteins and lipids coincide. For this scenario we turned to a reported specific interaction between the transmembrane protein p24 and N-stearoyl-sphingomyelin [9]. To this end, cells transiently transfected with a construct coding for Flag-tagged p24 were labeled with pacSph. As expected, labeling of p24 was observed both in HeLa and HeLa Δ*SGPL1* cells (Fig 6B), and competition with a 10fold molar excess of sphingosine lead to significant reduction in crosslink product. The labeling of proteins by pacSph is clearly dependent on the presence of UV radiation and pacSph in the medium (S15 Fig), which was efficiently incorporated into sphingolipids, as exemplarily shown for sphingomyelin (S16 Fig).

While the labeling in HeLa cells could be due to sphingolipids, but also to all other kinds of lipids derived from pac fatty acyl CoA that was generated by degradation pacSph, labeling in HeLa Δ*SGPL1* cells is restricted to sphingolipids only.

Therefore HeLa Δ*SGPL1* cells represent a valuable tool for studying protein sphingolipid interactions with sphingoid long chain base derived metabolic precursors.

## Conclusions

We have shown that *SGPL1* disruption is easily done in a cell line of choice by CRISPR-associated RNA-guided endonuclease Cas9 in a matter of weeks and propose that this should be easily transferable to other cells lines in which functionalized sphingoid bases are used [15, 19, 20], such as cell lines characterized by altered sphingolipid homeostasis. In summary, *SGPL1* knockout cell lines provide the basis to studying protein-sphingolipid interaction in physiological and pathophysiological states of cells.

## Supporting Information

S1 Equation Calculation of relative difference Δ—features. Relative changes of lipid features in the comparison of *Sgpl1*^(−/−)^ vs. *Sgpl1*^(+/+)^ in the MEF cell line and HeLa vs. HeLa Δ*SGPL1* cell line.
(PDF)

S1 Fig pac-Sphingosine (pacSph) a novel photoactivatable and clickable analog of sphingosine [15] that contains a photoactivatable diazirine ring and a terminal alkyne moiety. Diazirines can be activated by UV-light and cross-link to proteins in close proximity, while alkynes can be used in click chemistry, e.g. to link a fluorophore.
(PDF)

S2 Fig Simplified biosynthesis and degradation of sphingolipids (SP). pacSph (S1 Fig) or sphingosine (Sph, blue circle) and can either enter the biosynthetic pathway (green) yielding ceramide, sphingomyelin (SM) and glycosphingolipids (GSL) or the degradation pathway (red). The latter eventually produces palmityol-CoA that can also be incorporated into glycero- and glycerophospholipids like PC. S1P: sphingosine 1-phosphate, S1PL: S1P lyase, SK: sphingosine kinase, SPPase: S1P phosphatase, CerS: Ceramide synthases.
(PDF)

S3 Fig Standards for fluorescent TLC analysis (A) Standards purchased by Avanti, and N-(octadec-17-yn)- sphing-4-enin-1-phosphocholine (alkyne SM) subjected to click reaction with coumarin azide and separated on TLC. 0.5 nmol of each standard was used. (B) Synthesized alkyne SM used in different molar amounts for click reaction and TLC as in (A). (C) Positive ion mode precursor ion scanning, selected for fragment ions with m/z = 184, corresponding to the choline head group. Theoretical value: [M+H]^+^(alkyne SM) = 727.57 Da.
(PDF)

S4 Fig MEF *Sgpl1*^+/+^ and MEF *Sgpl1*^−/−^ Lipidome Analysis (A) Functional categories (B) Storage lipids standardized to all lipids without storage lipids, so they can be compared to Fig 1D. (C) Sphingolipid chain length distribution. (D) GPL chain length distribution. (E) Sphingolipid double bond distribution (F) Sphingolipid hydroxylation distribution. (F) GPL double bond distribution. A Welch two sample t-test was used to estimate the P values: *P < 0.05; **P < 0.01; *** P < 0.001. Error bars correspond to standard deviation (n = 6).
(PDF)

S5 Fig MEF *Sgpl1*^+/+^ and MEF *Sgpl1*^−/−^ sphingolipid species. Species are standardised to within each class. A Welch Two Sample t-test was used to estimate the p-values: * P < 0.05; ** P < 0.01; *** P < 0.001. Error bars correspond to standard deviation (n = 6). Species are shown as <lipid class> <number of carbon atoms>: <number of double bonds>; <number of hydroxyl groups>. Therefore SM 34:1;2 represents a sphingomyelin species with 34 carbon atoms, 1 double bond and 2 hydroxylations in the ceramide backbone.
(PDF)

S6 Fig Positioning of the *SGPL1* sgRNA Sequences (A) Human chromosome 10 and the position of *SGPL1* gene (uc001jrm.3) are shown. The gene model indicates all merged exons of in the USCF hg19 genome. S1–S4 indicate the position of sgRNA sequences chosen (See Table 1 in M&M). (B–D) Zoom of the exons of the targeted sgRNA sequences and their direction is indicated by blue arrows. Nucleotides indicated by colors: G (red), C (orange), T(blue), A (light blue). Created with R and the bioconductor R package Gvis and others [47–49].
(PDF)

S7 Fig Sequencing a HeLa Δ*SGPL1* clone (A) Alignment of sequence reads of the HeLa Δ*SGPL1*-S3 clone 4 with the wild type sequence. Reads have been found 3× and 4× respectively. (B) Alignment of sequence reads in the genomic context. Genomic changes were found at the position of the sgRNA sequence caused indel mutations often associated with frame shifts and stop codons.
(PDF)

S8 Fig Positioning of ALDH3A2 sgRNA (A) sgRNA sequences targeting ALDH3A2 inserted into the BbsI site of pSpCas9(BB)-2A-GFP. sgRNA sequences are indicated in blue and proceeded by a G nucleotide to improve transcription [29]. Oligos were designed with the CRISPR Design Tool (http://crispr.mit.edu) [29]. (B) Human chromosome 17 and the position of ALDH3A2 gene (uc002gwa.1) are shown. The gene model indicates exons in the USCF hg19 genome. A1–A4 indicate the position of sgRNA sequences chosen. (C) Zoom of the exon of the targeted sgRNA sequences and their direction by blue arrows. Nucleotides indicated by colors: G (red), C (orange), T (blue), A (light blue). Created with R and the bioconductor R package Gvis and others [47–49].
(PDF)

S9 Fig HeLa and HeLa Δ*SGPL1* lipidome analysis (A) Functional categories. (B) Storage lipids standardized to all lipids without storage lipids, so they can be compared to Fig 4. (C) Sphingolipid chain length distribution. (D) GPL chain length distribution. (E) Sphingolipid double bond distribution. (F) Sphingolipid hydroxylation distribution. (F) GPL double bond distribution. A Welch two sample t-test was used to estimate the P values: * P < 0.05; ** P < 0.01; *** P < 0.001. Error bars correspond to standard deviation (n = 3).
(PDF)

S10 Fig HeLa and HeLa Δ*SGPL1* sphingolipid species standardized to each class. A Welch Two Sample t-test was used to estimate the P values: * P < 0.05; ** P < 0.01; *** P < 0.001. Error bars correspond to standard deviation (n = 3). Species are shown as <lipid class> <number of carbon atoms>: <number of double bonds>; <number of hydroxyl groups>. Therefore SM 34:1;2 represents a sphingomyelin species with 34 carbon atoms, 1 double bond and 2 hydroxylations in the ceramide backbone.
(PDF)

S11 Fig Relative difference Δ-classes of MEF and HeLa cell. Relative changes of lipid classes in the comparison of *Sgpl1*^−/−^ to *Sgpl1*^+/+^ in the MEF cell line compared to HeLa Δ*SGPL1* and HeLa as calculated in S1 Equation.
(PDF)

S12 Fig Relative difference Δ-features of MEF and HeLa cells. Relative changes in the different features in the comparison of *Sgpl1*^(-/-)^ *Sgpl1*^(+/+)^ in the MEF cell line compared to HeLa Δ*SGPL1* and HeLa as calculated in S1 Equation. (A) Functional categories. (B) Storage lipids standardized to all lipids without storage lipids (C) Sphingolipid chain length distribution. (D) GPL chain length distribution. (E) Sphingolipid double bond distribution. (F) Sphingolipid hydroxylation distribution. (G) GPL double bond distribution.
(PDF)

S13 Fig CerS6 Immununoblot in Hela Δ*SGPL1*. HeLa and HeLa Δ*SGPL1* membranes were carbonate washed, floated and their proteins precipitated. An immunoplot for CerS6 is shown (CerS6, red). Detection of endogenous calnexin with anti-calnexin antibody (CNX, green) was used as a loading control. CerS6 (uniport: Q6ZMG9-1) has a predicted mass of 44.9 kDa.
(PDF)

S14 Fig Analysis of cell proliferation and apoptosis in HeLa and HeLa Δ*SGPL1* cells. A kinetic analysis of cell proliferation (A) and apoptosis (B) was conducted by quantifying cell confluence using an Essen BioScience IncuCyte Zoom live cell imaging microscope. For proliferation experiments the starting density measured by cell confluence was set to 100% for each experimental condition. The results shown are representative for three independent biological replicates, with 2000 (A) and 5000 (B) cells seeded per well. Error bars correspond to standard deviation (n = 3).
(EPS)

S15 Fig pac-Sph/UV Controls. Influence of pac-Sph labeling and UV-radiation on FLAG-p24 labeling in HeLa Δ*SGPL1* cells. Samples were treated as described in Fig 6.
(PDF)

S16 Fig Spectra of pacSph labeling. HeLa Δ*SGPL1* cells were labeled with 3 μM pacSph for 6 h, extracted, saponified, re-extracted and measured as described earlier [43]. Lipids with intensities greater 5% are indicated and shown in S7 Table.
(PDF)

S1 Table Quantification of pacSph fluorescence intensity in MEF *Sgpl1*^(+/+)^ and MEF *Sgpl1*^(−/−)^. The TLC is displayed in Fig 1A and data are plotted in Fig 1B. Individual values and Mean +/- SD (n = 3) are listed.
(XLSX)

S2 Table Quantification of fluorescence intensities in HeLa Δ*SGPL1* after pacSph metabolic labeling over a time course of 12 h and click reaction. The TLC is displayed in Fig 2C and plotted in Fig 2B. HeLa Δ*SGPL1* cells were labeled with 6 μM pacSph for up to 12 h. Lipids were extracted and subjected to click reaction with fluorogenic fluorescein, separated by TLC and exited with blue light. Lipids were identified via similarity to lipid standard (See Fig 2C). Individual values and Mean ± SD (n = 3) are shown.
(XLSX)

S3 Table Quantification of sphingoid bases and sphingoid base 1-phosphates. Data are plotted in Fig 4B. Individual values and Mean ± SD (n = 3) are shown.
(XLSX)

S4 Table Proteomics Screen: Differential Expression in HeLa and HeLa Δ*SGPL1*. Difference: Difference of log transformed LFQ (label free quantification) intensities of HeLa cells and HeLa Δ*SGPL1* cells (HeLa Δ*SGPL1* − HeLa). Statistical analysis was carried out with Perseus software. Significance Values are based on permuation based FDR analysis [44]: *: p < 0.05, **: p < 0.01. Data are plotted in Fig 5B.
(XLSX)

S5 Table Proteomics Screen: Proteins found only in HeLa cells. Protein LFQ intensities of HeLa cells where no intensity was found for HeLa Δ*SGPL1* cells. Only hits with high LFQ intensities in all replicates are shown. Data are plotted in Fig 5A.
(XLSX)

S6 Table Proteomics Screen: Proteins found only in HeLa Δ*SGPL1* cells. Protein LFQ intensities of HeLa Δ*SGPL1* cells where no intensity was found for HeLa cells. Only hits with high LFQ intensities in all replicates are shown. Data are plotted in Fig 5C.
(XLSX)

S7 Table Species assignment to pacSph labeled SM species. Species found in S16 Fig. Intensities greater 5% are shown.
(XLSX)

S1 Text Contains descriptive information regarding datasets and unmodified files available from Figshare: http://dx.doi.org/10.6084/m9.figshare.1449281. (PDF)

## Abbreviations

AcOH: acetic acid
CE: cholesterol esters
CHCl_3_: chloroform
CRISPR: clustered regularly interspaced short palindromic repeats
Cer: ceramide
Chol: cholesterol
DAG: diacylglycerol
DH-S1P: dihydrosphingosine-1-phosphate
GL: glycerolipid
GP: glycerophospholipid
GPL: glycerophospholipids and the glycerolipid DAG
HexCer: hexosylceramide
LFQ: label free quantification
MEF: mouse embryonic fibroblast
MEOH: methanol
NHEJ: non-homologous end joining
PC O-/PE O-: ether linked PC/PE
PC/PE/PG/PI/PS: phosphatidyl-choline/-ethanolamine/-glycerol/-inositol/-serine
PNS: post-nuclear supernatant
S1P: sphingosine-1-phosphate
SD: standard deviation
SGPL1: sphingosine-1-phosphate lyase 1 (Gene: *SGPL1*)
SM: sphingomyelin; SP, sphingolipid
ST: sterol; Spg, sphinganine
Spg1P: sphinganine-1-phosphate
Sph: sphingosine
TAG: triacylglycerol
TLC: thin-layer chromatography
pacSph: photoactivatable and clickable sphingosine
w/v: weight per volume
