# Supplementary material for: Sphingosine-1-Phosphate Lyase Deficient Cells as a Tool to Study Protein Lipid Interactions
Source: PLoS One. 2016 Apr 21;11(4):e0153009. doi: 10.1371/journal.pone.0153009 (PMC4839656; doi:10.1371/journal.pone.0153009)
Supplement: S1 Equation — Relative changes of lipid features in the comparison of Sgpl1(−/−) vs. Sgpl1(+/+) in the MEF cell line and HeLa vs. HeLa ΔSGPL1 cell line. (PDF) [file pone.0153009.s001.pdf]

$$\text{relative difference(Hela)} = \frac{feature_{Hela\Delta SGPL1} - feature_{Hela}}{feature_{Hela}} \tag{1}$$

$$\text{relative difference(MEF)} = \frac{feature_{Sgpl1(-/-)} - feature_{Sgpl1(+/+)}}{feature_{Sgpl1(+/+)}} \tag{2}$$
