# Supplementary figures and images for: Sphingosine-1-Phosphate Lyase Deficient Cells as a Tool to Study Protein Lipid Interactions
Source: PLoS One. 2016 Apr 21;11(4):e0153009. doi: 10.1371/journal.pone.0153009 (PMC4839656; doi:10.1371/journal.pone.0153009)

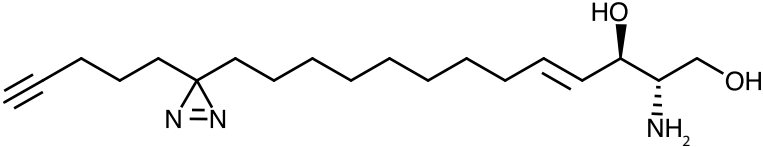

Supplement: S1 Fig — Diazirines can be activated by UV-light and cross-link to proteins in close proximity, while alkynes can be used in click chemistry, e.g. to link a fluorophore. (PDF) [file pone.0153009.s002.pdf]

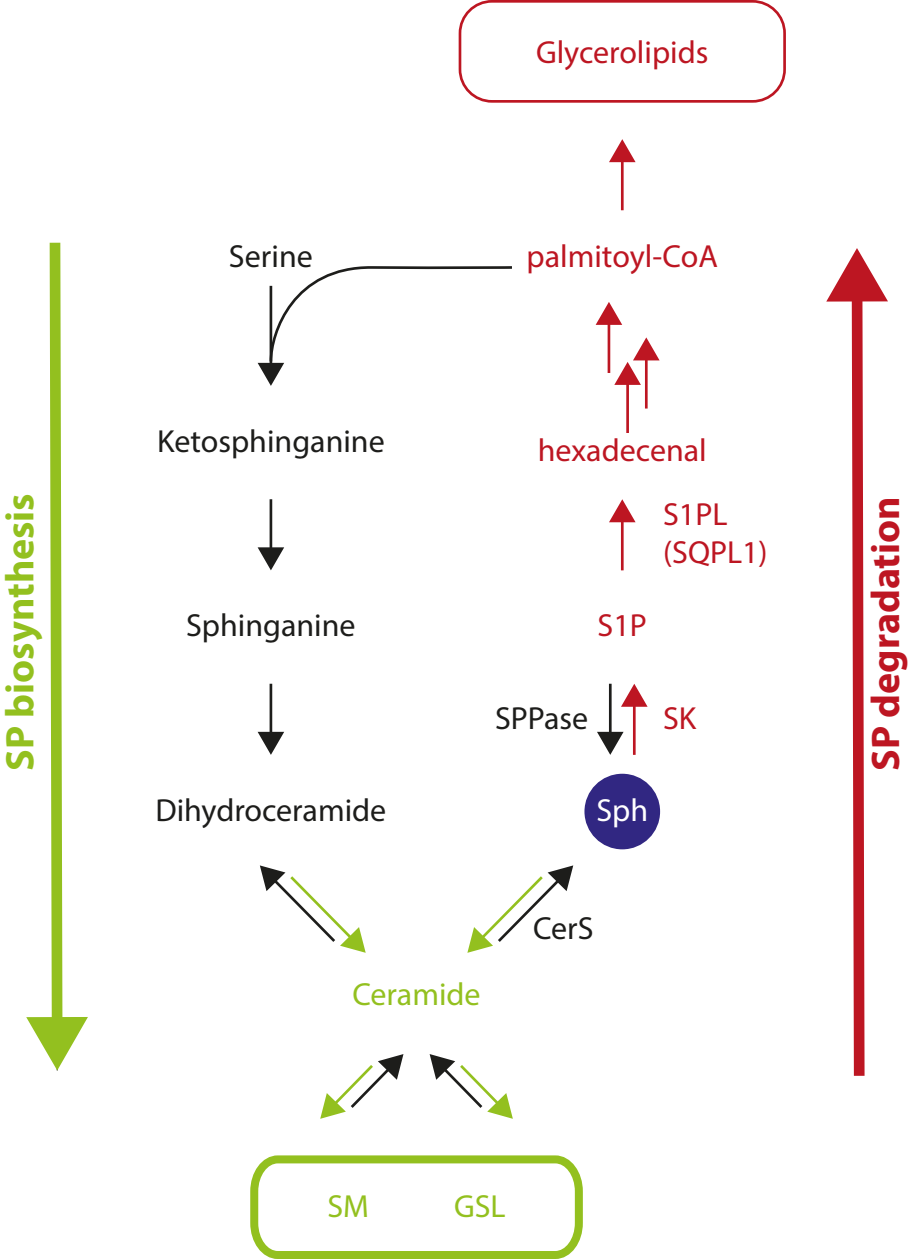

Supplement: S2 Fig — pacSph (S1 Fig) or sphingosine (Sph, blue circle) and can either enter the biosynthetic pathway (green) yielding ceramide, sphingomyelin (SM) and glycosphingolipids (GSL) or the degradation pathway (red). The latter eventually produces palmityol-CoA that can also be incorporated into glycero- and glycerophospholipids like PC. S1P: sphingosine 1-phosphate, S1PL: S1P lyase, SK: sphingosine kinase, SPPase: S1P phosphatase, CerS: Ceramide synthases. (PDF) [file pone.0153009.s003.pdf]

**A**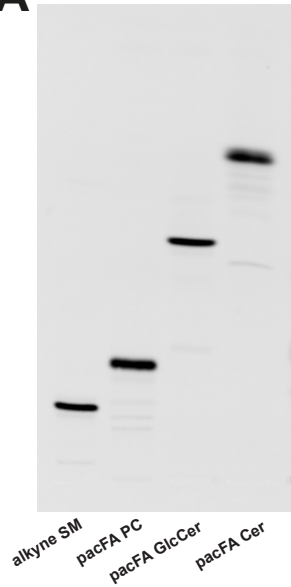**B**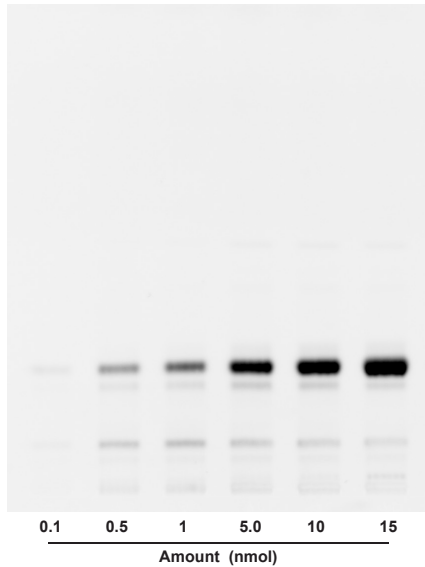**C**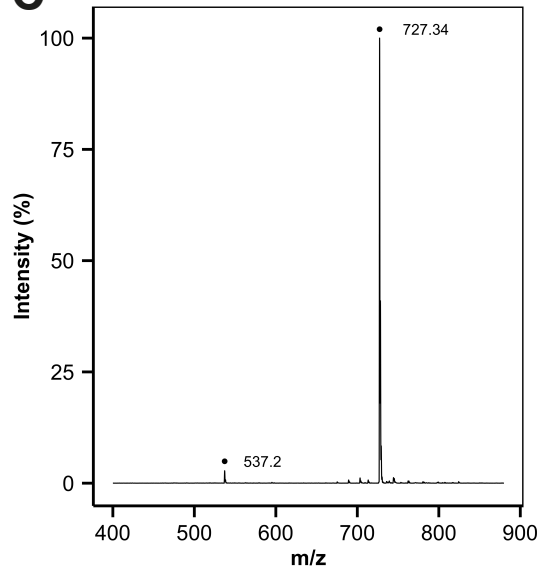

Supplement: S3 Fig — Standards for fluorescent TLC analysis (A) Standards purchased by Avanti, and N-(octadec-17-yn)- sphing-4-enin-1-phosphocholine (alkyne SM) subjected to click reaction with coumarin azide and separated on TLC. 0.5 nmol of each standard was used. (B) Synthesized alkyne SM used in different molar amounts for click reaction and TLC as in (A). (C) Positive ion mode precursor ion scanning, selected for fragment ions with m/z = 184, corresponding to the choline head group. Theoretical value: [M+H]+(alkyne SM) = 727.57 Da. (PDF) [file pone.0153009.s004.pdf]

**A**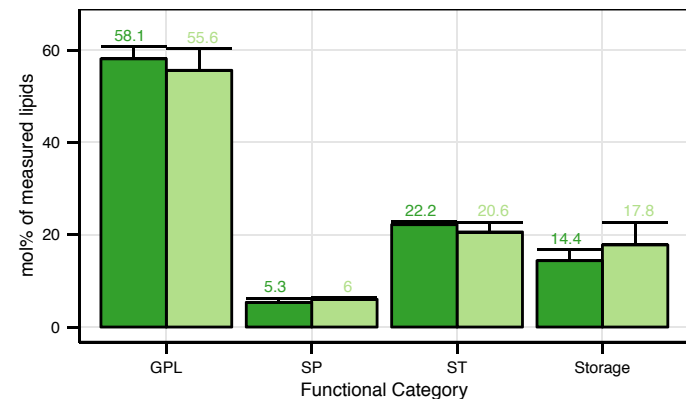**B**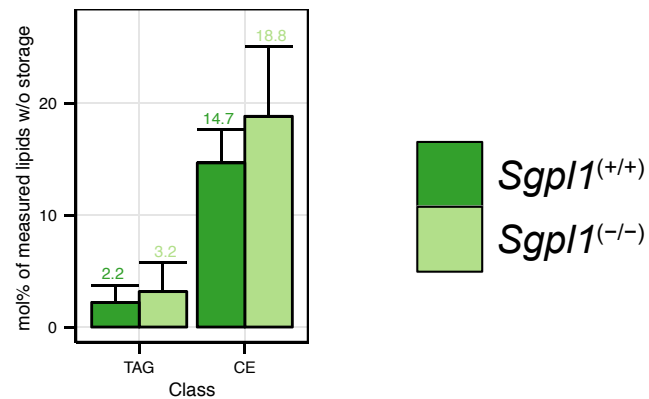**C**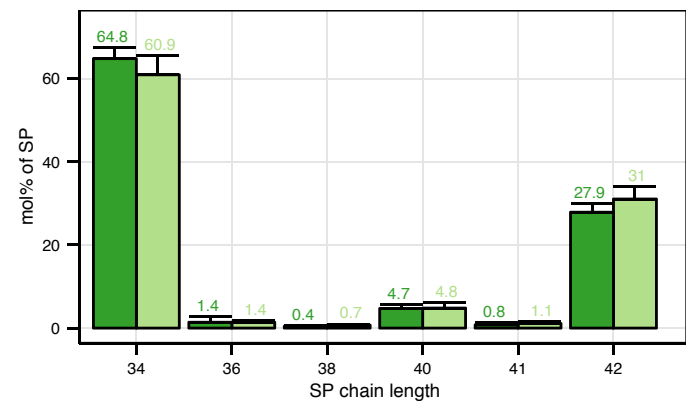**D**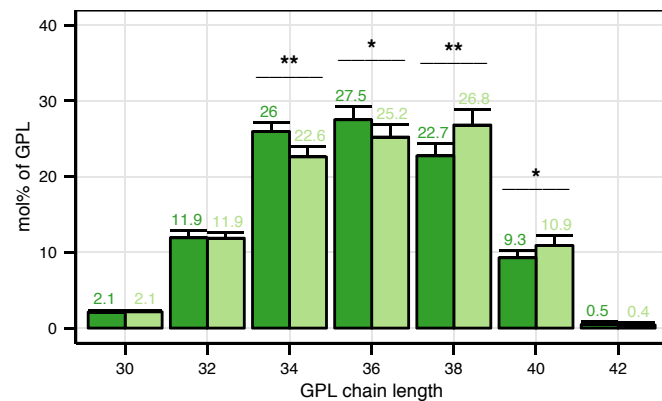**E**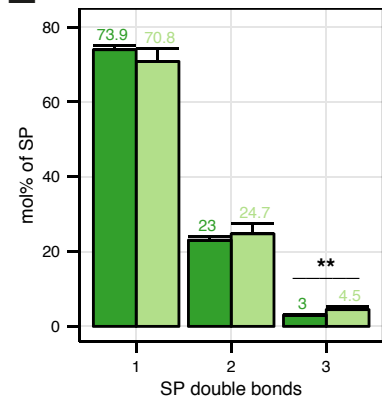**F**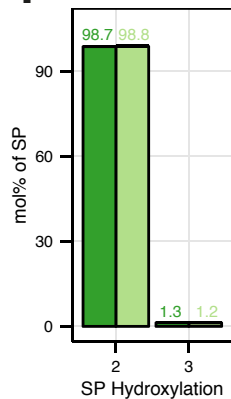**G**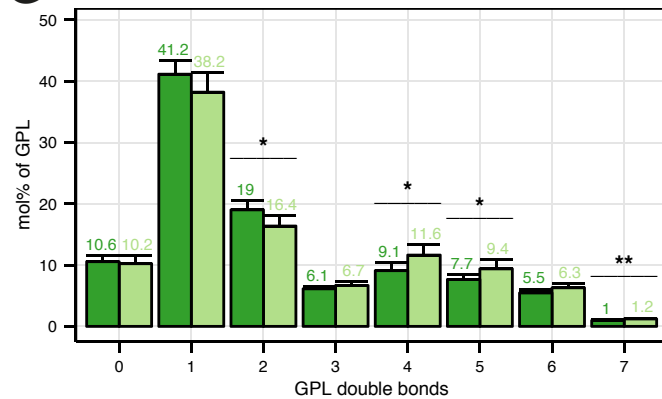

Supplement: S4 Fig — MEF Sgpl1+/+ and MEF Sgpl1−/− Lipidome Analysis (A) Functional categories (B) Storage lipids standardized to all lipids without storage lipids, so they can be compared to Fig 1D. (C) Sphingolipid chain length distribution. (D) GPL chain length distribution. (E) Sphingolipid double bond distribution (F) Sphingolipid hydroxylation distribution. (F) GPL double bond distribution. A Welch two sample t-test was used to estimate the P values: *P < 0.05; **P < 0.01; *** P < 0.001. Error bars correspond to standard deviation (n = 6). (PDF) [file pone.0153009.s005.pdf]

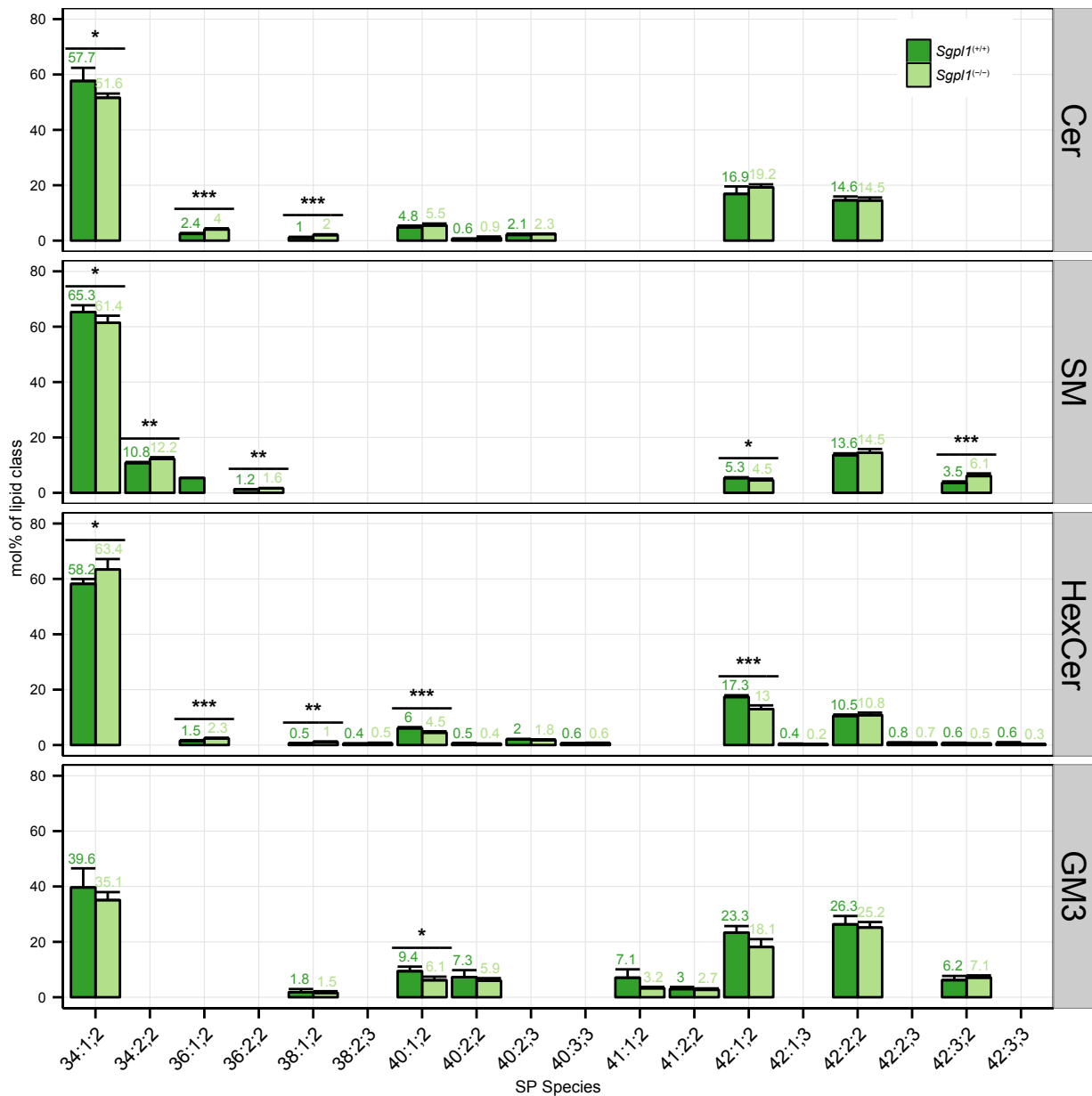

Supplement: S5 Fig — Species are standardised to within each class. A Welch Two Sample t-test was used to estimate the p-values: * P < 0.05; ** P < 0.01; *** P < 0.001. Error bars correspond to standard deviation (n = 6). Species are shown as : ; . Therefore SM 34:1;2 represents a sphingomyelin species with 34 carbon atoms, 1 double bond and 2 hydroxylations in the ceramide backbone. (PDF) [file pone.0153009.s006.pdf]

A

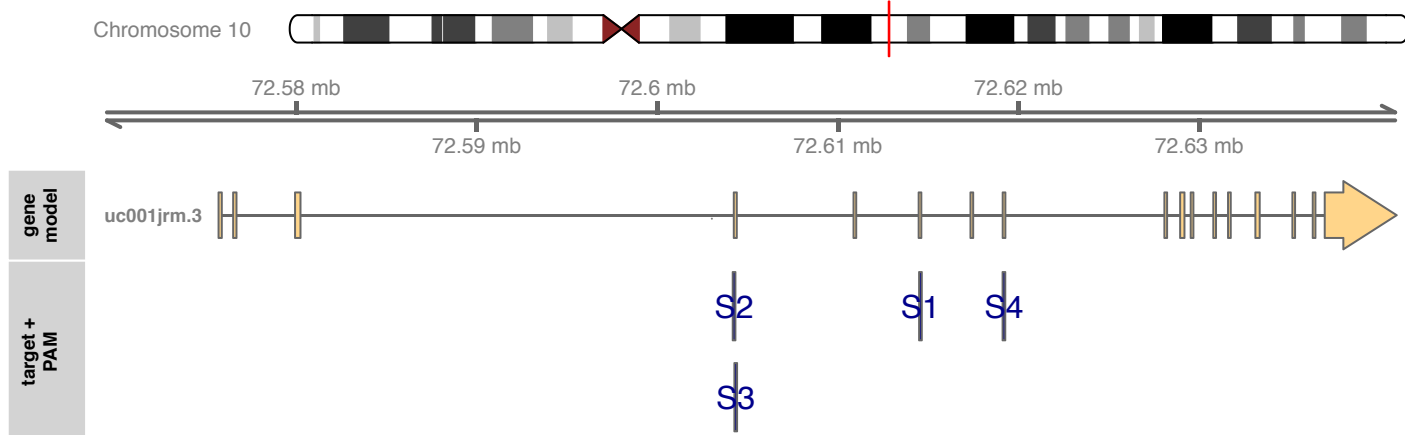

B

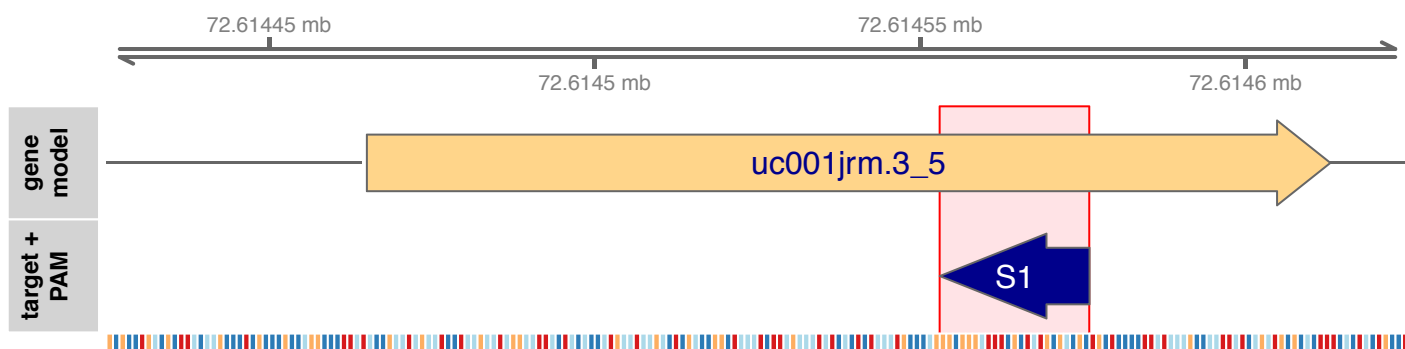

C

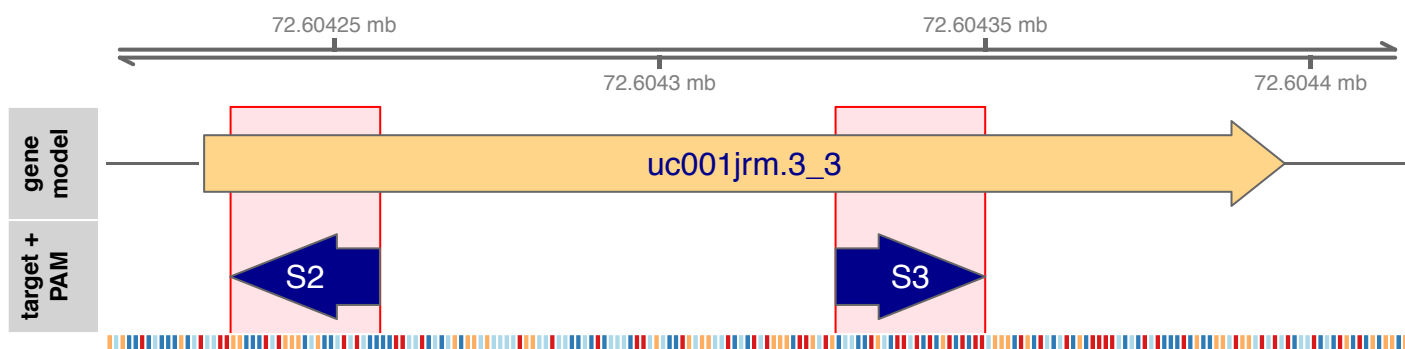

D

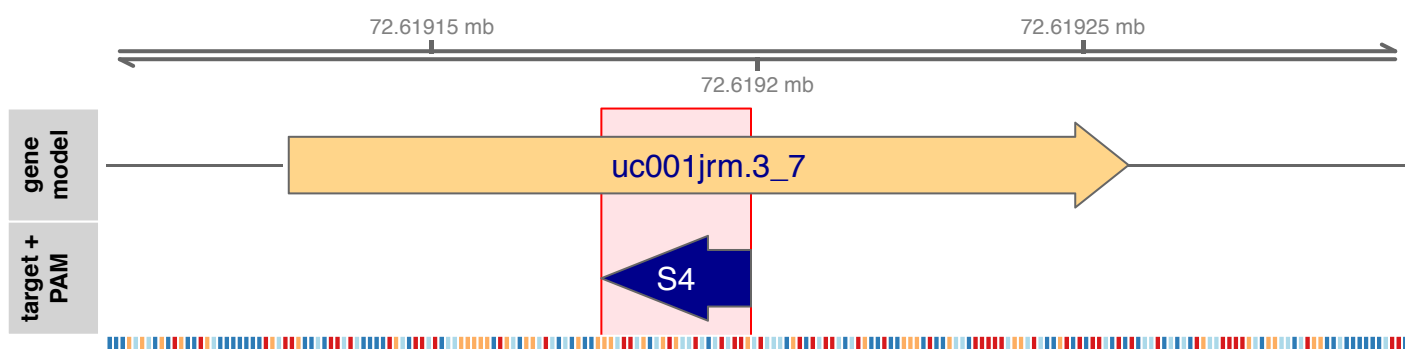

Supplement: S6 Fig — Positioning of the SGPL1 sgRNA Sequences (A) Human chromosome 10 and the position of SGPL1 gene (uc001jrm.3) are shown. The gene model indicates all merged exons of in the USCF hg19 genome. S1–S4 indicate the position of sgRNA sequences chosen (See Table 1 in M&M). (B–D) Zoom of the exons of the targeted sgRNA sequences and their direction is indicated by blue arrows. Nucleotides indicated by colors: G (red), C (orange), T(blue), A (light blue). Created with R and the bioconductor R package Gvis and others [47–49]. (PDF) [file pone.0153009.s007.pdf]

**A**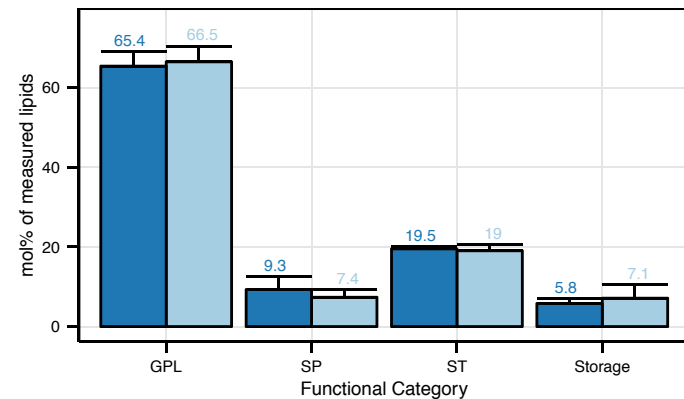**B**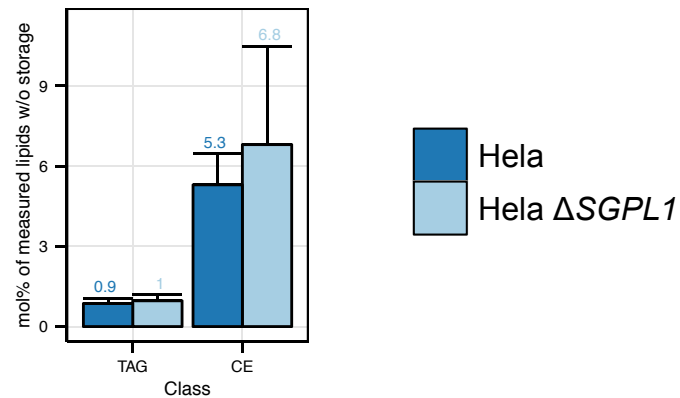**C**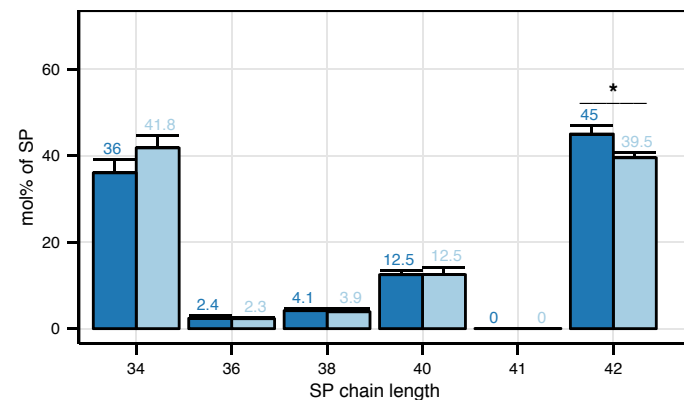**D**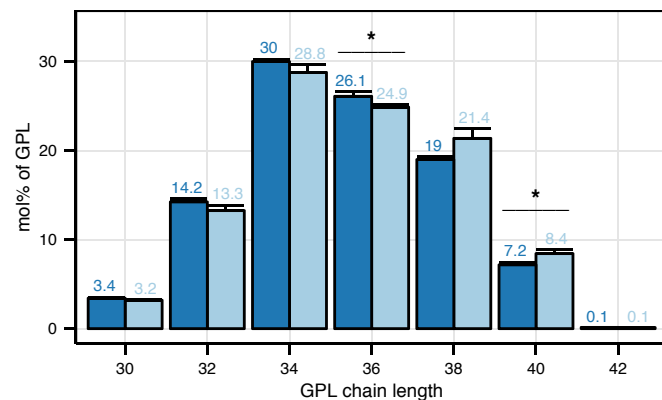**E**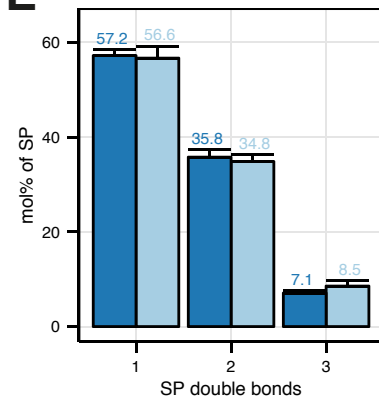**F**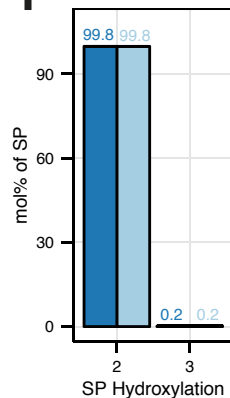**G**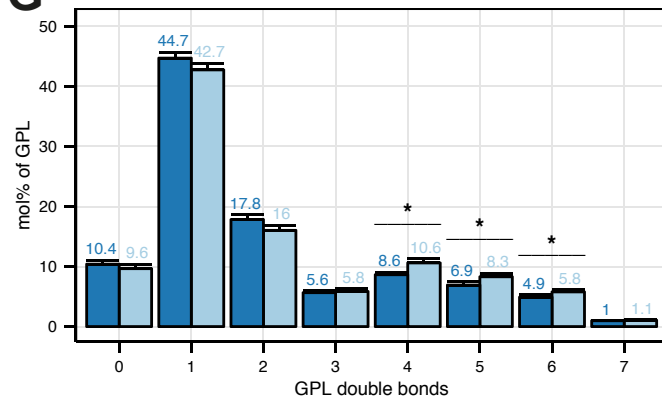

Supplement: S9 Fig — HeLa and HeLa ΔSGPL1 lipidome analysis (A) Functional categories. (B) Storage lipids standardized to all lipids without storage lipids, so they can be compared to Fig 4. (C) Sphingolipid chain length distribution. (D) GPL chain length distribution. (E) Sphingolipid double bond distribution. (F) Sphingolipid hydroxylation distribution. (F) GPL double bond distribution. A Welch two sample t-test was used to estimate the P values: * P < 0.05; ** P < 0.01; *** P < 0.001. Error bars correspond to standard deviation (n = 3). (PDF) [file pone.0153009.s010.pdf]

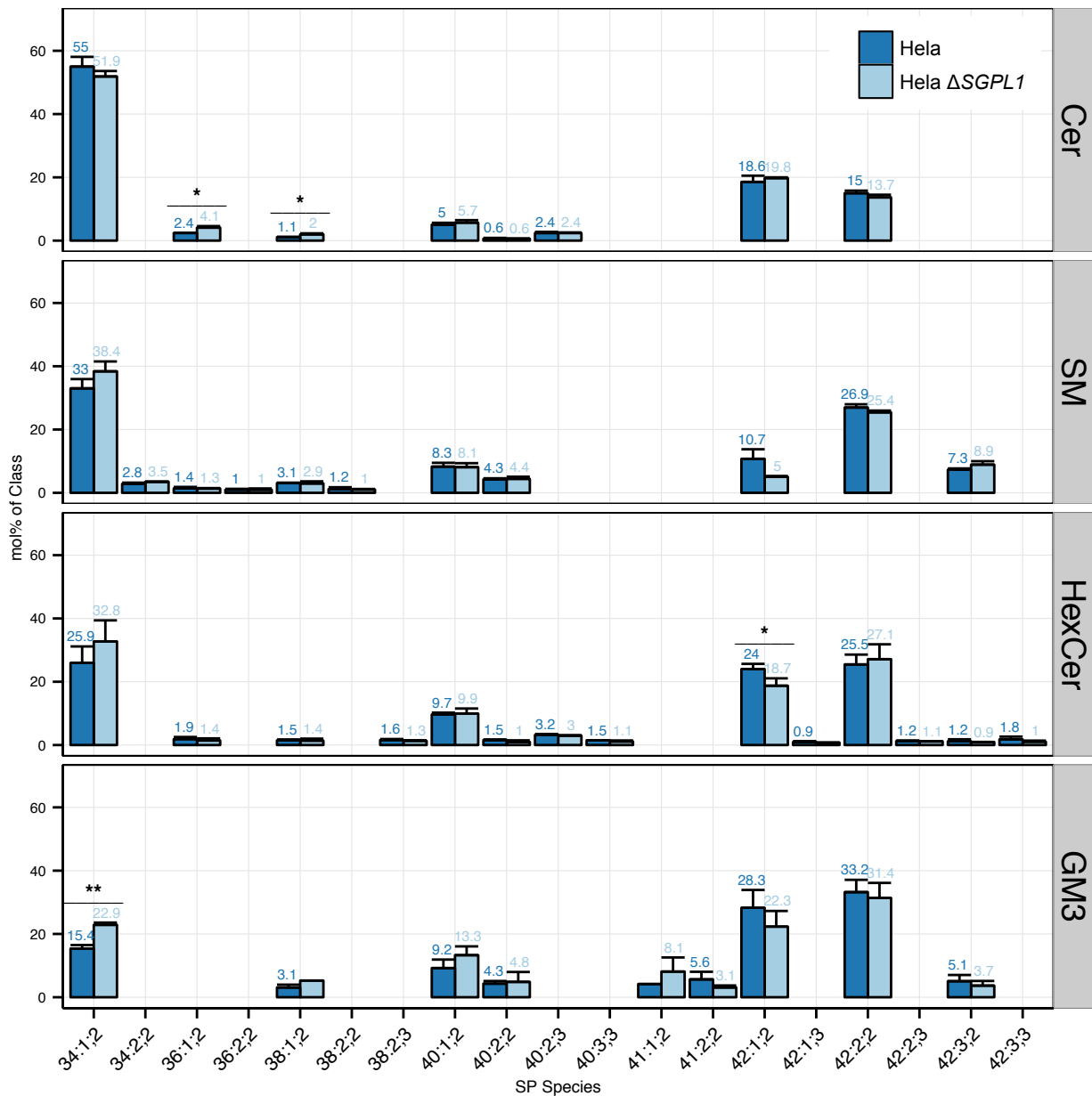

Supplement: S10 Fig — A Welch Two Sample t-test was used to estimate the P values: * P < 0.05; ** P < 0.01; *** P < 0.001. Error bars correspond to standard deviation (n = 3). Species are shown as : ; . Therefore SM 34:1;2 represents a sphingomyelin species with 34 carbon atoms, 1 double bond and 2 hydroxylations in the ceramide backbone. (PDF) [file pone.0153009.s011.pdf]

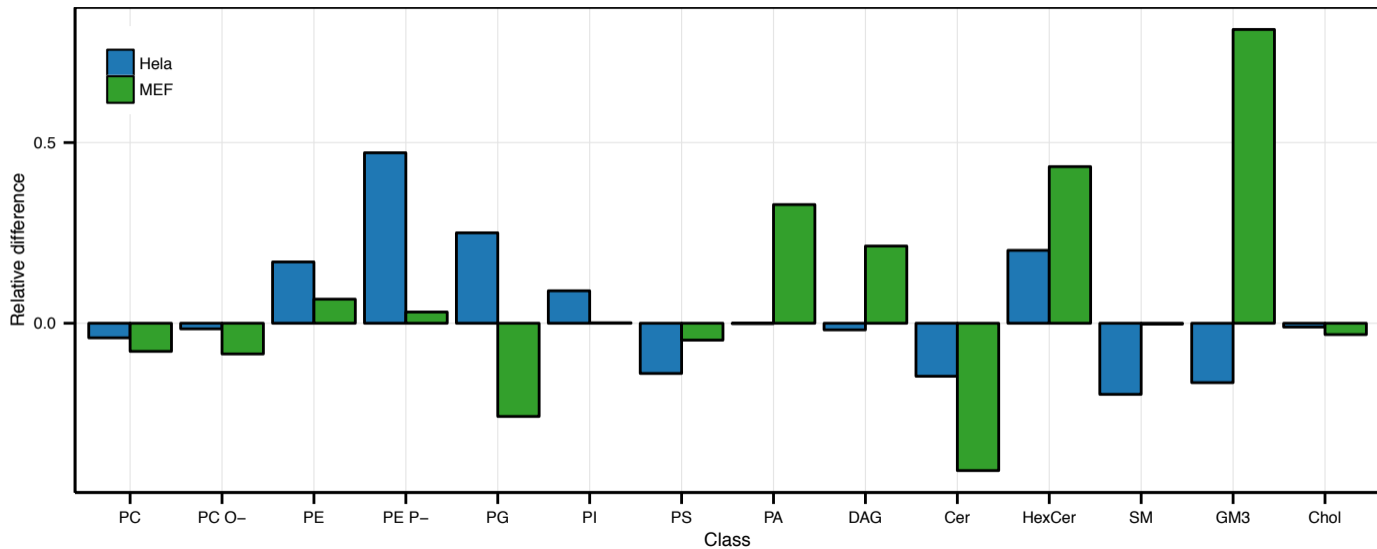

Supplement: S11 Fig — Relative changes of lipid classes in the comparison of Sgpl1−/− to Sgpl1+/+ in the MEF cell line compared to HeLa ΔSGPL1 and HeLa as calculated in S1 Equation. (PDF) [file pone.0153009.s012.pdf]

**A**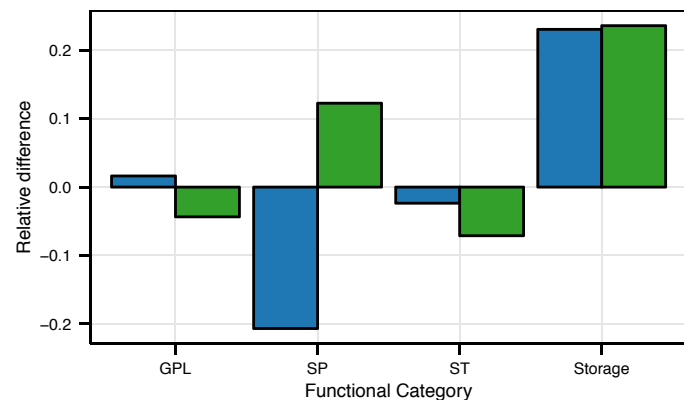**B**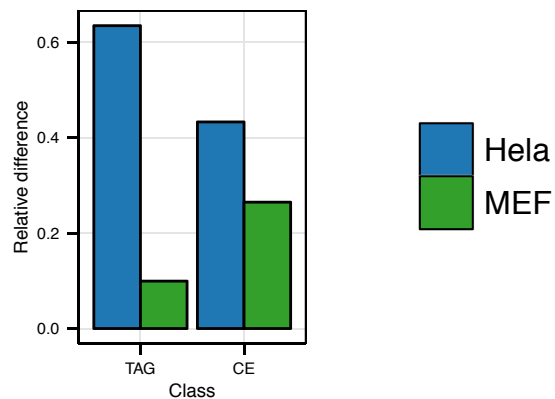**C**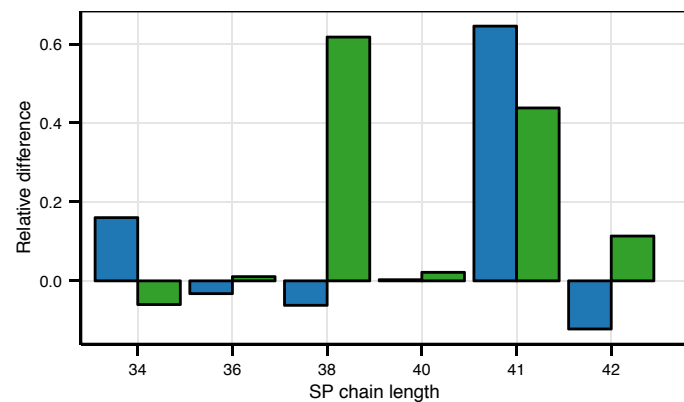**D**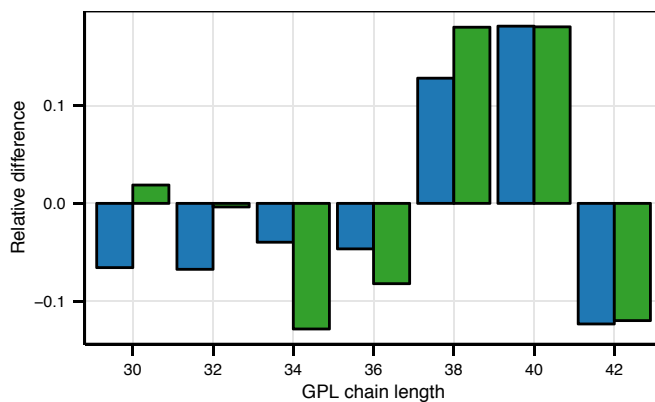**E**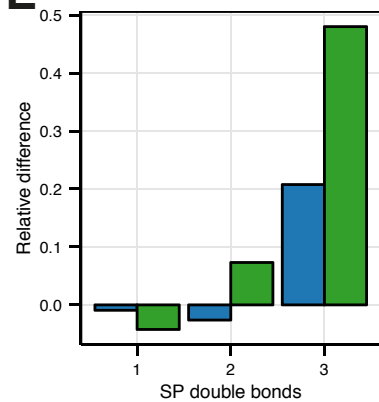**F**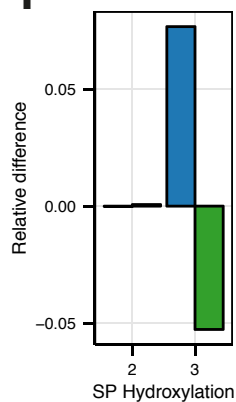**G**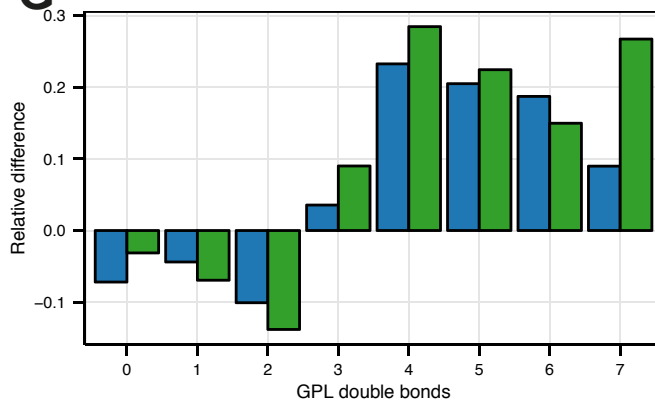

Supplement: S12 Fig — Relative changes in the different features in the comparison of Sgpl1(-/-) Sgpl1(+/+) in the MEF cell line compared to HeLa ΔSGPL1 and HeLa as calculated in S1 Equation. (A) Functional categories. (B) Storage lipids standardized to all lipids without storage lipids (C) Sphingolipid chain length distribution. (D) GPL chain length distribution. (E) Sphingolipid double bond distribution. (F) Sphingolipid hydroxylation distribution. (G) GPL double bond distribution. (PDF) [file pone.0153009.s013.pdf]

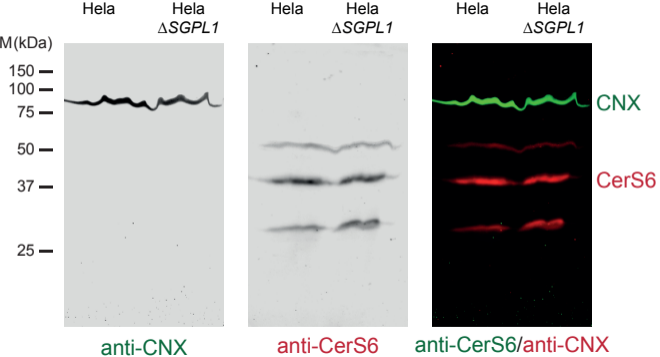

Supplement: S13 Fig — HeLa and HeLa ΔSGPL1 membranes were carbonate washed, floated and their proteins precipitated. An immunoplot for CerS6 is shown (CerS6, red). Detection of endogenous calnexin with anti-calnexin antibody (CNX, green) was used as a loading control. CerS6 (uniport: Q6ZMG9-1) has a predicted mass of 44.9 kDa. (PDF) [file pone.0153009.s014.pdf]

|               |   |   |   |
|---------------|---|---|---|
| <i>UV</i>     | — | + | + |
| <i>pacSph</i> | + | — | + |

|   |   |   |
|---|---|---|
| — | + | + |
| + | — | + |

|   |   |   |
|---|---|---|
| — | + | + |
| + | — | + |

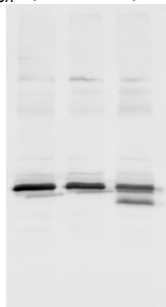

FLAG

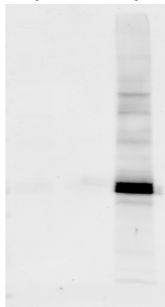

Alex647

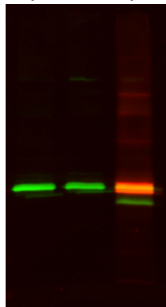

Overlay

Supplement: S15 Fig — Influence of pac-Sph labeling and UV-radiation on FLAG-p24 labeling in HeLa ΔSGPL1 cells. Samples were treated as described in Fig 6. (PDF) [file pone.0153009.s016.pdf]

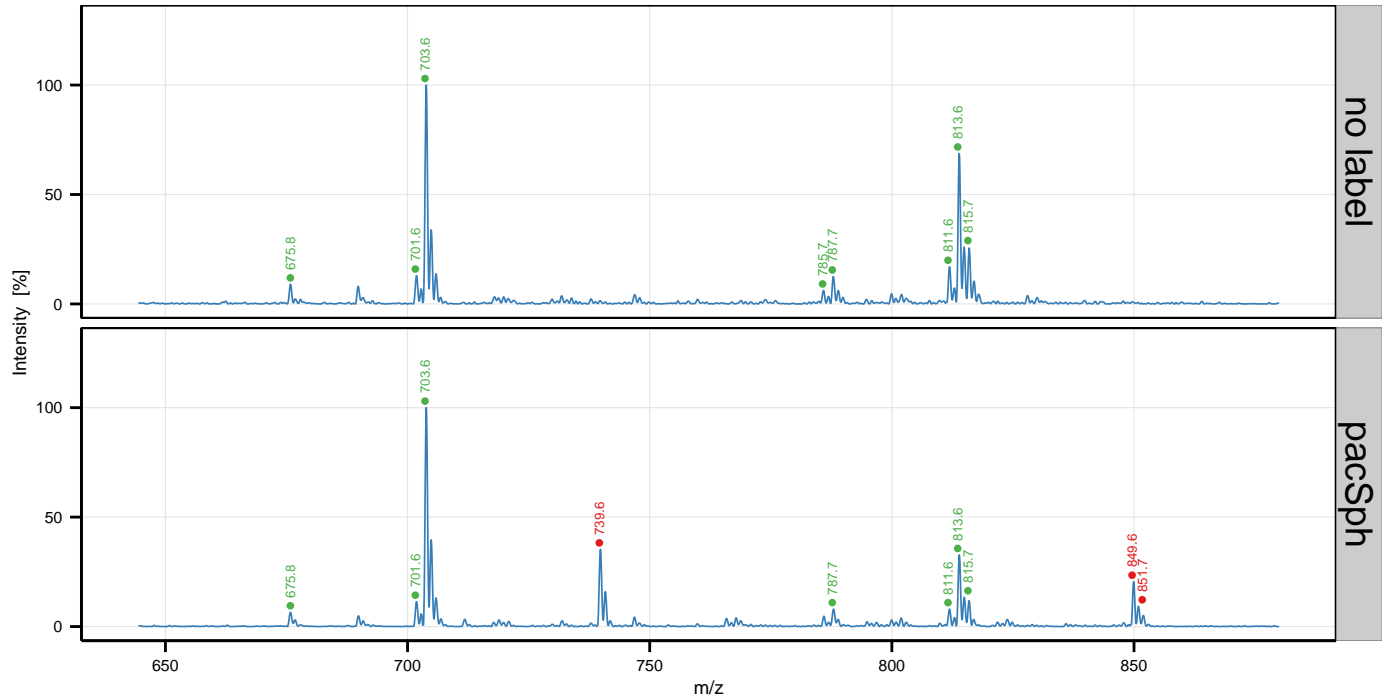

Supplement: S16 Fig — HeLa ΔSGPL1 cells were labeled with 3 μM pacSph for 6 h, extracted, saponified, re-extracted and measured as described earlier [43]. Lipids with intensities greater 5% are indicated and shown in S7 Table. (PDF) [file pone.0153009.s017.pdf]
