# Supplementary material for: Sphingosine-1-Phosphate Lyase Deficient Cells as a Tool to Study Protein Lipid Interactions
Source: PLoS One. 2016 Apr 21;11(4):e0153009. doi: 10.1371/journal.pone.0153009 (PMC4839656; doi:10.1371/journal.pone.0153009)

A

```
SQPL1      1>acattgcaccaagtatgagccctggcagctaattgcatggagtgt-cgtgtggaccctgctgatagtctggggatatgagtttgtcttccagccagag>97
S3.4_4x    1>ACATTGCACCAAGTATGAGCCCTGGCAGCTAAT---A-------T-CGTGTGGACCCTGCTGATAGTCTGGGGATATGAGTTTGTCTTCCAGCCAGAG>87
S3.4_3x    1>ACATTGCACCAAGTATGAGCCCTGGCAGCTAATTGCATGGAGTGTCCGTGTGGACCCTGCTGATAGTCTGGGGATATGAGTTTGTCTTCCAGCCAGAG>98
```

B

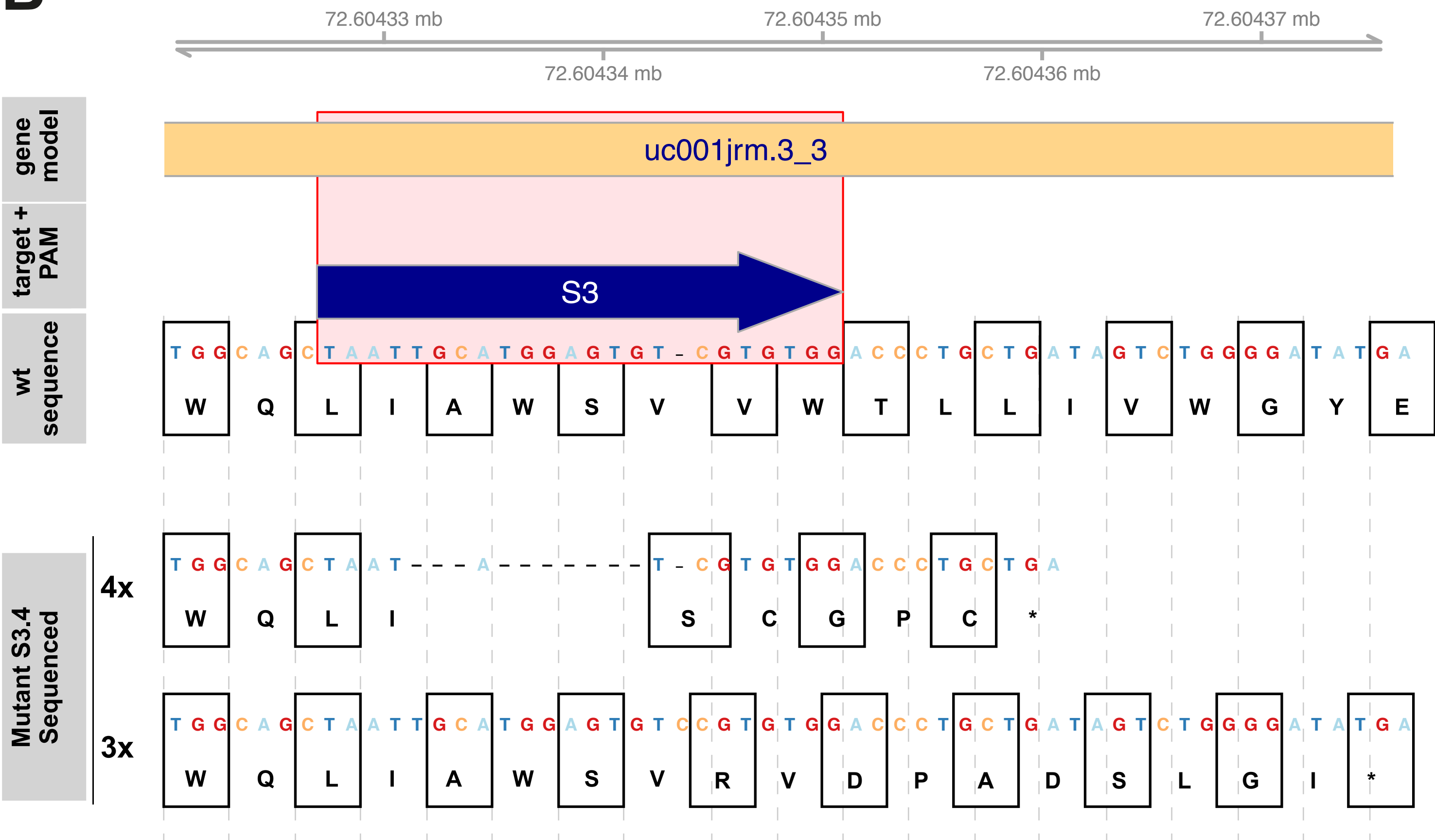

Supplement: S7 Fig — Sequencing a HeLa ΔSGPL1 clone (A) Alignment of sequence reads of the HeLa ΔSGPL1-S3 clone 4 with the wild type sequence. Reads have been found 3× and 4× respectively. (B) Alignment of sequence reads in the genomic context. Genomic changes were found at the position of the sgRNA sequence caused indel mutations often associated with frame shifts and stop codons. (PDF) [file pone.0153009.s008.pdf]
