# Supplementary material for: Sphingosine-1-Phosphate Lyase Deficient Cells as a Tool to Study Protein Lipid Interactions
Source: PLoS One. 2016 Apr 21;11(4):e0153009. doi: 10.1371/journal.pone.0153009 (PMC4839656; doi:10.1371/journal.pone.0153009)

A

sgRNA	Orientation	Sequence
A1	fwd	CACCGCGAAGTCCGGCGGGTCCGAC
A1	rev	AAACGTCGGACCCGCCGGACTTCGC
A2	fwd	CACCGGAAACCGCAGAGGTCGCGAC
A2	rev	AAACGTCGCGACCTCTGCGGTTTCC
A3	fwd	CACCGGGTCGGCGGCGATGGCCGTC
A3	rev	AAACGACGGCCATCGCCGCCGACCC
A4	fwd	CACCGGATGGTGCAGGAGCGCGAGA
A4	rev	AAACTCTCGCGCTCCTGCACCATCC

B

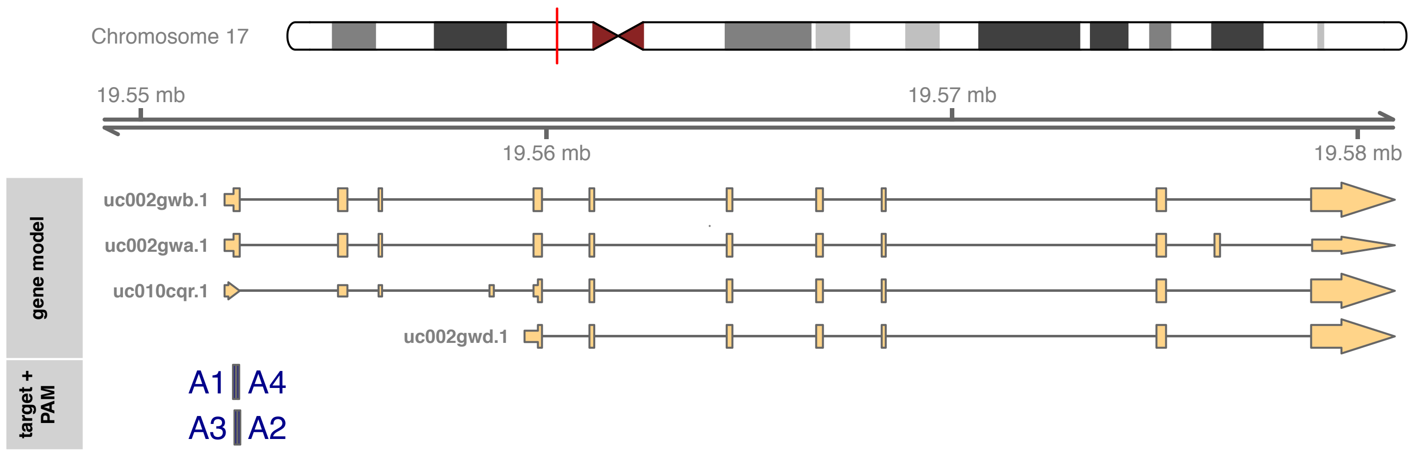

C

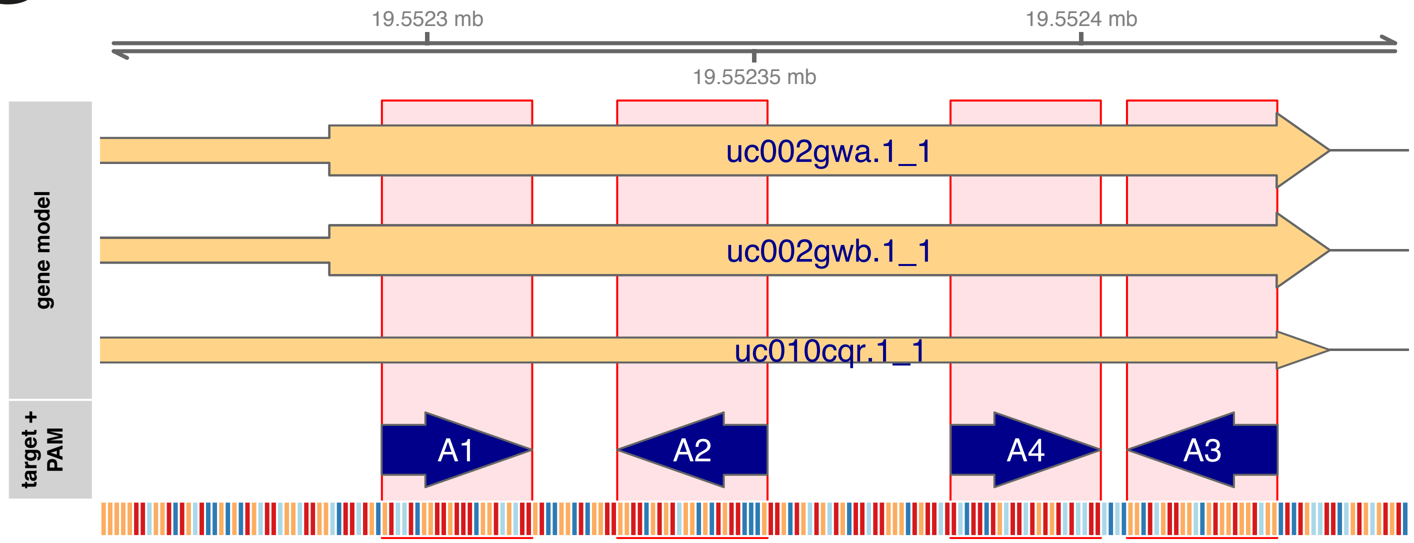

Supplement: S8 Fig — Positioning of ALDH3A2 sgRNA (A) sgRNA sequences targeting ALDH3A2 inserted into the BbsI site of pSpCas9(BB)-2A-GFP. sgRNA sequences are indicated in blue and proceeded by a G nucleotide to improve transcription [29]. Oligos were designed with the CRISPR Design Tool (http://crispr.mit.edu) [29]. (B) Human chromosome 17 and the position of ALDH3A2 gene (uc002gwa.1) are shown. The gene model indicates exons in the USCF hg19 genome. A1–A4 indicate the position of sgRNA sequences chosen. (C) Zoom of the exon of the targeted sgRNA sequences and their direction by blue arrows. Nucleotides indicated by colors: G (red), C (orange), T (blue), A (light blue). Created with R and the bioconductor R package Gvis and others [47–49]. (PDF) [file pone.0153009.s009.pdf]
