# Supplementary material for: Sphingosine-1-Phosphate Lyase Deficient Cells as a Tool to Study Protein Lipid Interactions
Source: PLoS One. 2016 Apr 21;11(4):e0153009. doi: 10.1371/journal.pone.0153009 (PMC4839656; doi:10.1371/journal.pone.0153009)
Supplement: S1 Text — (PDF) [file pone.0153009.s025.pdf]

Datasets and Original Data for:

Sphingosine-1-phosphate lyase deficient cells as a tool to study protein lipid interactions

Mathias J. Gerl[1], Verena Bittl[1], Susanne Kirchner[1], Timo Sachsenheimer[1], Hanna L. Brunner[1], Christian Lüchtenborg[1],* Cagakan Özbalci[1], *Hannah Wiedemann[1], Sabine Wegehingel[1], Walter Nickel[1], Per Haberkant[2], Carsten Schultz[2], Marcus Krüger[3] and Britta Brügger[1*]*

[1] Heidelberg University Biochemistry Center, Heidelberg, Germany.

[2] European Molecular Biology Laboratory, Heidelberg, Germany.

[3] CECAD, Cologne, Germany.

*Corresponding authors

Email: Mathias.Gerl@bzh.uni-heidelberg.de (MJG);

Britta.bruegger@bzh.uni-heidelberg.de (BB)

Data in Repository

All datasets and unmodified files are available from Figshare:

http://dx.doi.org/10.6084/m9.figshare.1449281

Datasets:

S1 Dataset. Complete dataset of the lipid composition the MEF *Sgpl1*$^{+/+}$ and *Sgpl1*$^{-/-}$ cells. Data given as mol% of total lipids in 6 replicates.

S2 Dataset. Complete dataset of the lipid composition of HeLa and for HeLa Δ*SGPL1* cells**.** Data given as mol% of total lipids in 3 replicates.

Original Data

S1 Unmodifed Image. Fluorescent TLC of Fig. 1A. *Sgpl1*$^{+/+}$ and *Sgpl1*$^{-/-}$ MEF cells were metabolically labeled with 6 µM pacSph for 4 h. Lipids were extracted and subjected to click reaction with fluorogenic coumarin azide, separated by TLC and exited with UV light.

S2 Unmodifed Image. Fluorescent TLC of Fig. 1C. *Sgpl1*$^{+/+}$ and *Sgpl1*$^{-/-}$ MEF cells were metabolically labeled with 6 µM pacSph for 4 h. Lipids were extracted, subjected to alkaline hydrolysis or mock treatment, followed by click reaction with fluorogenic coumarin azide, separated by TLC and exited with UV light.

S3 Unmodifed Image. Fluorescent TLC of Fig. 2A. Characterization of the HeLa Δ *SGPL1* cell lines. HeLa wildtype (**H**, Lane 1) and HeLa Δ *SGPL1* (**Δ** *,* lane 2) cells were

labeled with 6 µM pacSph for 6 h. Lipids were extracted and subjected to click reaction with fluorogenic fluorescein, separated by TLC and excited with blue light.

S4 Unmodifed Image. Fluorescent TLC of Fig. 2C. Characterization of the HeLa Δ *SGPL1* cell lines. HeLa *ΔSGPL1* cells were labeled with 6 µM pacSph for the time points indicated in Fig. 2C. Lipids were extracted and subjected to click reaction with fluorogenic fluorescein, separated by TLC and excited with blue light. As reference, 50 pmol of each clickable Cer, HexCer, PC and SM (S3 Fig.) was loaded as lipid standard.

S5 Unmodifed Image. Fluorescent TLC of Fig. 3. Rescue of HeLa Δ *SGPL1* cells by exogenous Flag-SGPL1. HeLa wildtype, HeLa Δ *SGPL1* and HeLa Δ *SGPL1* transfected with Flag-tagged SGPL1 were labeled with 6 µM pacSph for 4 h. Extracted lipids were subjected to click reaction with fluorogenic coumarin azide, separated by TLC and exited with UV light. Lipids were identified via with alkyne lipid standards (S3 Fig) seen on left. Samples displayed. Replicate 1 [HeLa wildtype, HeLa Δ *SGPL1* and HeLa Δ *SGPL1* transfected], Replicate 2 [HeLa wildtype, HeLa Δ *SGPL1* and HeLa Δ *SGPL1* transfected], HeLa Δ *SGPL1* -S3 clone 4 [control, transfected].

S6 Unmodifed Image. Immunoblot Files of Fig. 3. Rescue of HeLa Δ *SGPL1* cells by exogenous Flag-SGPL1. HeLa wildtype, HeLa Δ *SGPL1* and HeLa Δ *SGPL1* transfected with Flag-tagged SGPL1 were labeled with 6 µM pacSph for 4 h. Proteins precipitated during the lipid extraction were solubilized, analyzed by SDS-PAGE and

western blotting, using anti-Flag antibodies (700 nm). Detection of endogenous calnexin with anti-calnexin antibody (800 nm) was used as a loading control. Samples displayed. Replicate 1 [HeLa wildtype, HeLa Δ*SGPL1* and HeLa Δ*SGPL1* transfected], Replicate 2 [HeLa wildtype, HeLa Δ*SGPL1* and HeLa Δ*SGPL1* transfected], HeLa Δ*SGPL1* -S3 clone 4 [control, transfected]. Molecular weight marker: Precision Plus Protein All Blue Standard (Bio-Rad 161-0373).

S7 Unmodifed Image. Immunoblot Files of Fig. 6A. Fluorescent labeling of pacSph metabolites to study protein lipid interaction. Flag-tagged proteins were expressed in HeLa and HeLa Δ*SGPL1* cell lines. After metabolic labeling with pacSph for 7 h, cells were UV irradiated to cross-link pacSph metabolites to nearby protein. Protein lysates were subjected to click reaction with Alexa647 azide (700 nm) and the ectopically expressed proteins were immunoprecipitated. After SDS-PAGE and immunoblot with fluorescently labeled secondary antibodies (800 nm), lipid and protein signals were detected in separate channels. STARD7: 0008462_01, p24: 0008463_01. Molecular weight marker: Precision Plus Protein All Blue Standard (Bio-Rad 161-0373).

S8 Unmodifed Image. Fluorescent TLC of Fig. S3A. Standards for fluorescent TLC analysis. Standards purchased by Avanti, and N-(Octadec-17-yn)- Sphing-4-enin-1-Phosphocholine (alkyne SM) subjected to click reaction with coumarin azide and separated on TLC.

S9 Unmodifed Image. Fluorescent TLC of Fig. S3B. Standards for fluorescent TLC analysis. Different amounts of N-(Octadec-17-yn)- Sphing-4-enin-1-Phosphocholine (alkyne SM) were subjected to click reaction with coumarin azide and separated on TLC.

S10 Unmodifed Image. Immunoblot Files of Fig. S13. HeLa and HeLa Δ*SGPL1* membranes were carbonate washed, floated and their proteins precipitated. An immunoplot for CerS6 is shown (CerS6, 700 nm). Detection of endogenous calnexin with anti-calnexin antibody (CNX, 800nm) was used as a loading control.

Molecular weight marker: Precision Plus Protein All Blue Standard (Bio-Rad 161-0373).

S11 Unmodifed Image. Immunoblot Files of Fig. S15. pac-Sph/UV Controls. Influence of pac-Sph labeling and UV-radiation on FLAG-p24 labeling in HeLa Δ*SGPL1* cells. Channels: Alex647 (700 nm), anti-FLAG (800 nm). Molecular weight marker: Precision Plus Protein All Blue Standard (Bio-Rad 161-0373).

S12 Unmodified Image. Immunoblot Files of Fig 6B. Fluorescent labeling of pacSph metabolites to study protein lipid interaction. WB of HeLa samples: Overlay of red and green channel: 20160205_HeLa_p24_original, WB of HeLa Δ*SGPL1* samples: Overlay of red and green channel: 20160205_HeLaS1PL_p24_original.
